# 3D heterospecies spheroids of pancreatic stroma and cancer cells demonstrate key phenotypes of pancreatic ductal adenocarcinoma

**DOI:** 10.1016/j.tranon.2021.101107

**Published:** 2021-05-01

**Authors:** Xinyuan Liu, Beate Gündel, Xidan Li, Jianping Liu, Anthony Wright, Matthias Löhr, Gustav Arvidsson, Rainer Heuchel

**Affiliations:** a Pancreas Cancer Research Lab, Department of Clinical Science, Intervention and Technology, (CLINTEC), Karolinska Institutet, Huddinge SE 141 86, Sweden; b Department of Medicine, Karolinska Institutet, Huddinge SE 141 86, Sweden; c Division of Biomolecular and Cellular Medicine, Department of Laboratory Medicine, Karolinska Institutet, Huddinge SE 141 86, Sweden

**Keywords:** Pancreatic cancer, Cancer associated fibroblasts, Co-culture, Spheroids, Tumor microenvironment, 3D, three dimensional, ABC, ATP-binding cassette, a-SMA, alpha smooth muscle actin / ACTA2, actin alpha 2, smooth muscle, CAFs, cancer-associated fibroblasts, ccK18, caspase-cleaved cytokeratin 18, DEGs, differentially expressed genes, ECM, extracellular matrix, EMT, epithelial mesenchymal transition, FBS, fetal bovine serum, FOLFIRINOX (a combination of folinic acid/leucovorin, 5-FU, irinotecan and oxaliplatin), GEM, gemcitabine, GSEA, gene set enrichment analysis, HMGCR, 3-hydroxy-3-methylglutaryl coenzyme A reductase, iCAFs, inflammatory CAFs, IFNα, interferon alpha, IFNγ, interferon gamma, IHC, immunohistochemistry, imPSCc2, immortalized mouse pancreatic stellate cells clone 2, ITS-A, insulin-transferrin-selenium-sodium-pyruvate solution, LDLR, low-density lipoprotein receptor, MSigDB, molecular signatures database, mTORC1, mammalian target of rapamycin complex 1, myCAFs, myofibroblastic CAFs, NF-κB, nuclear factor kappa B, NuMA, nuclear mitotic apparatus protein, PAC, paclitaxel, PCSK9, proprotein convertase subtilisin/kexin type 9, PDAC, pancreatic ductal adenocarcinoma, PSCs, pancreatic stellate cells, qRT-PCR, quantitative Real-Time PCR, RPKM, Reads Per Kilobase of Transcript per Million Reads Mapped, scRNA-seq, single-cell RNA sequencing, Shh, sonic hedgehog, TCGA, the cancer genom atlas, TEM, transmission electron microscopy, TGF-β1, transforming growth factor-beta1, TGFBR1/2, TGF-β receptor type I/II, TMM, trimmed mean of M-values, TNF, tumor necrosis factor

## Abstract

Pancreatic ductal adenocarcinoma (PDAC) is one of the most lethal malignancies, partly due to the dense desmoplasia and a lack of suitable model systems to study. In the present work, we developed a 3D heterospecies spheroid model to study the microenvironmental interactions between tumor cells and stellate cells which can also be employed to test therapeutic regimens. We set up monospheroids and heterospheroids made up from murine pancreatic stellate cells (mPSCs) and human PDAC cells (Panc1), which allowed for direct isolation of mRNA from a mixed cell population followed by an *in silico* separation of the RNA-seq reads. Global transcript level changes for cells in heterospheroids versus monospheroids were calculated, followed by gene set enrichment analysis and molecular subtype analysis. We observed an apparent shift of Panc1 from the classical to the squamous/basal-like phenotype upon co-culture with mPSCs. Moreover, mPSCs acquired a different cancer-associated fibroblast-related phenotype upon co-culture with Panc1. We analyzed the tumor cell-specific chemosensitivities towards gemcitabine, paclitaxel and SN38 and compared these to published pharmacotranscriptomic signatures. In conclusion, our heterospecies spheroid model reflected key aspects of PDAC and facilitated the study of intercellular interactions between tumor and stroma while additionally proving to be a good model for studying therapeutic responses.

## Highlights

• We described a straightforward and highly reproducible 3D culturing method to investigate the intercellular crosstalk between pancreatic cancer and pancreatic stellate cells (PSCs)/cancer associated fibroblasts (CAFs).
• Pancreatic ductal adenocarcinoma (PDAC) is therapy resistant and characterized by a desmoplastic microenvironment with low functional vascularization. As a consequence, this cancer type is nutrition-poor.
• Using expression profiling, we compared the common culturing condition using ten percent fetal bovine serum (FBS) with 0.1percent FBS and found that PDAC cells when grown together with PSCs/CAFs change into a more aggressive subtype, independent of the FBS concentration in the medium.
• Depending on the FBS concentration, the PSCs/CAFs display a shift from a myofibroblastic CAF (myCAF) to a more inflammatory CAF (iCAF) phenotype in co-culture with PDAC cells.
• We demonstrate that the pharmacotranscriptomic signatures as well as the chemoresistance of the PDAC cells change significantly upon co-culture with PSCs/CAFs.

## Introduction

Pancreatic ductal adenocarcinoma (PDAC) is one of the most lethal solid malignancies worldwide [1]. A lack of diagnostic biomarkers in combination with advanced disease at the time of diagnosis and an almost complete resistance of cancer cells to conventional chemo- and radiation therapy regimens are the major reasons for the poor clinical outcome [2]. Therefore, pancreatic cancer should be treated as a medical emergency [3].

Gene mutation and mRNA expression analyses on bulk tumor tissue have been performed by several labs to stratify PDAC into cellular subtypes [4], [5], [6]. Two major classes have been described: a squamous subtype (including quasi-mesenchymal, basal-like and squamous) that is more aggressive, and a classical subtype (including pancreatic progenitor, classical, and aberrantly differentiated endocrine exocrine) [7]. The idea behind these stratification efforts have been to correlate the molecular subtypes to prognosis and clinical treatment response.

One of the hallmarks of PDAC is the dense desmoplastic stroma, which is triggered by PDAC cells secreting transforming growth factor-beta1 (TGF-β1) [8]. It is characterized by excessive extracellular matrix (ECM) deposition, high abundance of cancer-associated fibroblasts (CAFs), infiltration of leukocytes and endothelial cell recruitment [9]. CAFs are responsible for promoting tumor progression, modulating and facilitating tumor metastasis and angiogenesis, influencing drug access and therapy response [10]. Recently, two subtypes of CAFs with different roles were discovered by Öhlund et al [11]. CAFs that locate proximal to the cancer buds and express elevated levels of alpha-smooth muscle actin (a-SMA, Acta2) are considered as myofibroblastic CAFs (myCAFs), whereas the other CAF subtype which locates more distal and secretes proinflammatory cytokines is referred to as inflammatory CAFs (iCAFs) [11]. Activated pancreatic stellate cells (PSCs) are the major source of CAFs in PDAC [12], and the crosstalk between PSCs/CAFs and tumor cells has attracted much attention [13].

Compared with traditional monolayer cell culture, three-dimensional (3D) cell culture systems more closely resemble *in vivo* conditions within a microenvironment, characterized by gradients for nutrients, oxygen and metabolic waste products [14]. Established cell lines grown under 3D culture conditions are usually defined as spheroids that can produce ECM and show central hypoxia as well as increased chemo-resistance when compared to monolayer culture systems [15,16]. Spheroids are highly uniform and more reproducible than organoids, which are derived directly from primary tissues. For PDAC research, we and others have developed advanced spheroid culture approaches, where the cancer cells were combined with cell types from the tumor stroma, such as fibroblasts, macrophages or pancreatic stellate cells [17], [18], [19]. A general limitation of previous investigations has been the difficulty to investigate the crosstalk between different cell types without physical separation of differentially labelled cells or by single-cell RNA sequencing (scRNA-seq). In both cases, dissociation and cell sorting is needed prior to RNA extraction and RNA-seq library preparation. These lengthy procedures can lead to changes in gene expression [20]. In order to circumvent the single cell preparation step, we generated heterospecies heterospheroids consisting of mouse PSCs and human tumor cells and subsequently used an *in silico* approach for deconvoluting RNA-seq short reads based on species origin from the mixed cell fraction [21]. This approach, which was initially developed for deconvoluting RNA-seq short reads from xenograft samples has since been successfully used in mixed-species co-cultures of cell lines [22].

In the present work, the spheroids were cultured under common cell culture growth factor rich condition with 10% fetal bovine serum (FBS), hereafter called high serum condition. The intratumoral vasculature of PDAC, however, is compressed and has low functionality due to the dense and ECM-rich stroma [12,23]. This problem together with the extremely high content of collagenous extracellular matrix makes diffusion difficult and results in a tumor microenvironment that is poor in nutrients and growth factors [24]. To mimic the nutrient/growth factor deprived environment, we also set up the spheroid cultures supplemented with only 0.1% FBS, hereafter called low serum condition.

With this advanced 3D co-culture model based on murine PSCs and human PDAC cells, we were able to study gene expression changes influenced by the intercellular crosstalk between tumor and stroma under different culture conditions by species-specific transcriptomic analysis.

## Materials and methods

### Monolayer Cell culture

The well characterized human pancreatic cancer cell line Panc1 was purchased from ATCC [25]. The immortalized mouse PSC clone 2 (imPSCc2), hereafter called mouse PSC (mPSC), was a kind donation from Dr. Raul Urrutia and Dr. Angela Mathison at the Mayo Clinic College of Medicine, Rochester, Minn, USA [26]. Panc1 and mPSCs were cultivated either under high serum condition with DMEM/F12 (Gibco 31330095) medium supplemented with 10% FBS (Gibco 10270106) and 0.5% penicillin/streptomycin (Gibco 15070063) [19] or low serum condition supplemented with 0.1% FBS, 0.3% bovine serum albumin (only for Panc1 cells) (Sigma A9647), insulin-transferrin-selenium-sodium pyruvate solution (ITS-A) (Gibco 51300044; 1/10 of the recommended concentration was used to adapt insulin to more physiological concentrations) and 0.5% penicillin/streptomycin in a humidified incubator at 37°C and 5% CO_2_. Both Panc1 and mPSCs were stepwise adapted to low serum condition over a course of 6–8 weeks. Absence of mycoplasma was checked regularly by MycoAlertTM PLUS Mycoplasma Detection Kit (LT07-705, Lonza, Switzerland).

### Spheroid formation assay

Panc1 and mPSCs were trypsinized and grown either as monospheroids (2500 mPSCs or Panc1 cells) or heterospheroids (mPSCs and Panc1 at different ratios, 2500 cells in total) in non-treated, round-bottom 96-well microplates (Falcon, 351177, BD NJ, USA) in DMEM/F12 medium supplemented with either 10% FBS (“high serum” condition) or 0.1% FBS, 0.3% BSA and ITS-A (“low serum” condition), and 0.5% penicillin/streptomycin as well as 0.24% methyl cellulose at 37°C and 5% CO_2_ humidified condition [16,19]. Growth curves were generated by quantifying spheroid viability using the CellTiter-Glo® 3D Cell Viability Assay (Promega, Germany).

### Spheroid preparation for immunohistochemistry (IHC)

Spheroids were collected following 5 days of cultivation, washed in phosphate buffered saline and fixed in 4% paraformaldehyde for 1 h at room temperature. Then spheroids were transferred to biopsy cryomolds (Tissue-Tek Cryomold #4565). About 100 µl HistoGel (ThermoFisher Scientific, HG-4000-012) was used to embed spheroids at one corner of the cryomold, followed by addition of another 300 µl HistoGel to fill up the biopsy cryomold. Then, the biopsy cryomold was put on ice to solidify the gel. After that, the solid gel was gently transferred into a biopsy cassette and kept in 70% ethanol until further processing [16,19]. Paraffin-embedded spheroids were sectioned at 4 μm before hematoxylin-eosin staining or immunohistochemistry for human specific nuclear mitotic apparatus protein (NuMA, Abcam, #ab84680) at the Morphological Phenotype Analysis (FENO) core facility at Karolinska Institutet. Panc1 and mPSCs in the sections were counted using ImageJ [27].

### Transmission electron microscopy (TEM)

Monospheroids and heterospheroids under high and low serum condition were collected and fixed by 2.5% glutaraldehyde and 1% formaldehyde in 0.1 M phosphate buffer [16,19]. Then the spheroids were rinsed in 0.1 M phosphate buffer prior to postfixation with 2% osmium tetroxide in 0.1 M phosphate buffer, pH 7.4 at 4 °C for 2 h. The spheroids were then stepwise dehydrated in ethanol, followed by acetone and finally embedded in LX-112. Ultrathin sections (~50–60 nm) were prepared using an EM UC7 (Leica) and were contrasted with uranyl acetate followed by lead citrate. TEM imaging was done in a Hitachi HT7700 transmission electron microscope (Hitachi High-Technologies) operated at 80 kV and digital images were acquired using a Veleta CCD camera (Olympus Soft Imaging Solutions) at the electron microscopy unit of Karolinska Institutet.

### RNA extraction, library preparation and sequencing

Five days after seeding, spheroids were collected, and total RNA was extracted directly from monospheroids and heterospheroids for RNA-sequencing using the RNeasy PLUS kit (Qiagen). Samples were quality assessed and quantified by RNA screen on TapeStation (Agilent) and Qubit (Thermo Fisher) dsDNA assay. Library preparations were conducted using NEBNext® Ultra™ II Directional RNA Library Prep Kit (NEB, E7760S), including a poly-A enrichment step by the NEBNext® Poly(A) mRNA Magnetic Isolation Module (NEB, E7490S). The End Repaired DNA was subjected to Agencourt AMPure XP bead clean-up and size selection (NEB, E6260). Libraries from the samples were multiplexed using NEBNext® Multiplex Oligos for Illumina® (NEB, Index Primers Set 1; E7335S) and sequenced in one lane using an Illumina HiSeq3000 instrument at the Integrated Cardio Metabolic Centre at Karolinska Institutet, generating on average 20 million single-end 50 bp reads per sample.

### Species based read separation and mapping to reference genomes

Reference genomes for human and mouse, GRCh38.87 and GRCm38.87 were downloaded from Ensembl and used to build respective indices for the species-based read classifier Xenome (1.0.1) [21] and the short read aligner STAR (2.5.1b) [28]. The program Xenome was used to classify the fastq reads based on species origin using default settings [21]. Separated human and murine reads were subsequently aligned to their respective reference genomes by means of STAR using default options with –sjdbGTFfile pointing to the respective reference annotation file and –outFilterMultimapNmax set to 1 [28]. featureCounts from the package subread (1.5.2) was employed to quantify reads mapped to known features in the Ensembl database [29].

### **Differential expression analysis, gene set enrichment analysis (GSEA) and molecular subtype analys**i**s**

Differential gene expression analysis between heterospheroids and monospheroids under high serum and low serum condition was conducted by edgeR (v3.30.3) using the glmQLF workflow [30]. Genes with different transcript level in heterospheroids compared to monospheroids with an FDR q-value < 0.05 and an absolute fold change > 1.5 were considered as differentially expressed genes (DEGs). The EnhancedVolcano (v1.6.0) R package was used to visualize transcript level differences [31]. Venn diagrams were generated using the R package VennDiagram (v1.6.20) [32]. GSEA (v4.0.3) [33] was conducted for Panc1 heterospheroids vs. monospheroids, as well as mPSC heterospheroids vs. monospheroids in both high serum and low serum conditions, respectively. Trimmed mean of M-values (TMM) normalized RPKM (Reads Per Kilobase of transcript per Million mapped reads) expression levels were used for GSEA. Gene sets of hallmark (h.all.v7.1.entrez.gmt) [34] and KEGG subsets from canonical pathways (c2.cp.kegg.v7.2.symbols.gmt) [35], downloaded from the molecular signatures database (MSigDB), were included in the analysis. Gene set sizes fewer than 10 and more than 500 were excluded from analysis. The DEGs of Panc1 were compared to the classical/basal-like signatures [5], progenitor/squamous signatures [6] and chemosensitivity signatures [36] using Fisher's exact test. The DEGs of mPSCs were compared to CAF signatures [11] using Fisher's exact test. Expression levels were visualized by the pheatmap (v1.0.12) R package [37].

### The cancer genome atlas (TCGA) cohort analysis

The normalized RNA expression data and clinical data in TCGA_PAAD project [38] https://portal.gdc.cancer.gov/projects/TCGA-PAAD were downloaded using the TCGAbiolinks R package (v2.16.4) [39]. Subtype information for the Moffitt's and Bailey's classification schemes were extracted using the same tool. The TCGAbiolinks edgeR-powered function was used to determine DEGs for the Moffitt's classification (basal-like vs. classical) and the Bailey's classification (squamous vs. progenitor), respectively [39]. FDR *q*-value < 0.05 and absolute fold change > 1.5 were applied as cutoffs. The difference between the proportions of the DEGs with higher transcript levels for Panc1 grown in heterospheroids and the DEGs with higher transcript levels for Panc1 grown in monospheroids that overlapped with the DEGs with higher transcript levels from patients in the TCGA cohort based on Moffitt's classification and Bailey's classification were interrogated by Fisher's exact test.

### Quantitative real-time PCR (qRT-PCR)

Total RNA of monospheroids and heterospheroids was isolated and 0.25 µg of total RNA was reverse transcribed to synthesize cDNA by the iScript cDNA Synthesis kit (Bio-Rad, 1708891) [19]. Species-specific primers for semiquantitative RT-PCR were designed based on the sequence differences between mouse and human homologues using PRIMER3 (v.0.4.0) [40]. Species specificity of the primers (Supplemental Table S1) was verified by testing each primer pair on cDNA preparations from both human and murine cell lines. The qRT-PCR reaction was performed using Thermo Scientific™ Maxima SYBR Green/Fluorescein qPCR Master Mix kit (ThermoFisher Scientific, K0243) [19]. RPL13A/Rpl13a for human and mouse served as housekeeping genes, respectively. For each gene, three independent biological replicates were performed. Statistical analyses based on delta CT values were conducted by Student's t-test (2-sided, individual samples). Gene expression was calculated by 2^−ΔΔct^ method.

### Drug treatments and apoptosis assay

Monospheroids and heterospheroids were seeded as described before [16,19], and after one day, treated with 5 µM TGF-β receptor type I/II (TGFBR1/2) kinase inhibitor (LY2109761, Sigma-Aldrich, #SML2051) or different therapeutic compounds, including 50 µM gemcitabine (Sigma-Aldrich, #G6423), 1 µM paclitaxel (Sigma-Aldrich, #580555), 1 µM SN38 (active metabolite of irinotecan; Sigma-Aldrich, #H0165) or 5 µM pitavastatin (a HMG-CoA reductase inhibitor; Selleckchem, #S1759). Three days later, spheroids were collected for RNA extraction or apoptosis detection. M30 Apoptosense® CK18 Kit (Diapharma #P10011) was used according to the manufacturer's instructions to quantitatively detect apoptosis in epithelial cells and differences between treatment groups were assessed by paired t-test.

## Results

### Establishment of a simple heterospecies heterospheroid cell culture system

**Fig. 1:**
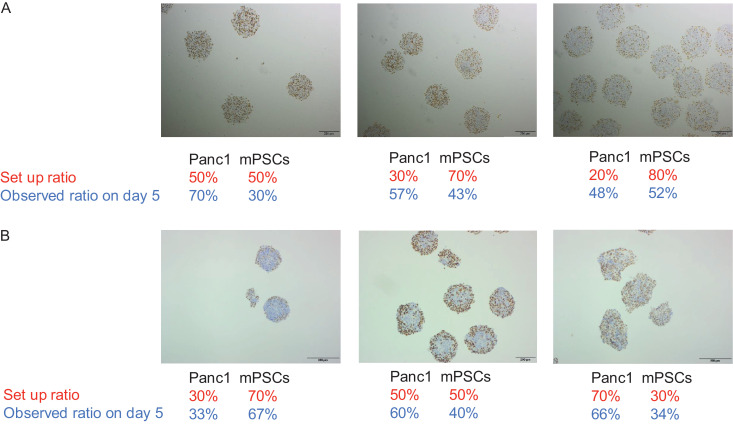
Co-cultures of murine PSCs and human pancreatic cancer cells (A) NuMA staining of Panc1/mPSCs heterospheroids grown in high serum condition for 5 days. (B) Analogous to A for cells grown in low serum condition. Panc1 and mPSCs were seeded in different ratios, 2500 cells in total, as indicated in the figure. Brown color shows NuMA positive cells (Panc1), whereas blue color shows mPSCs. Size bar: 200 µm.

In order to find the optimal seeding ratio, Panc1 and mPSCs were co-cultured at different ratios in high serum (50:50, 30:70 and 20:80 Panc1: mPSCs) and low serum (30:70, 50:50 and 70:30 Panc1: mPSCs) for 5 days. Thereafter, the cellular composition of the spheroids was assessed by human specific NuMA staining (Fig. 1A and B). Panc1 and mPSCs seeded in a ratio of 20:80 (500 Panc1 cells and 2000 mPSCs) under high serum and 50:50 (1250 Panc1 cells and 1250 mPSCs) under low serum condition resulted in approximately equal cell numbers following 5 days of co-culture. These seeding ratios were used in all subsequent experiments. The growth kinetics of Panc1, mPSCs monospheroids and heterospheroids grown under high serum and low serum conditions are shown in Supplemental Figure S1A and S1B. As expected, Panc1 monospheroids and Panc1/mPSCs heterospheroids under high serum condition grew faster compared to low serum condition. TEM images show the morphology of Panc1, mPSCs monospheroids and heterospheroids on day 5 under high serum and low serum condition (Supplemental Figure S1C).

### The transcriptional changes of Panc1 cells co-cultured with mPSCs in spheroids represented *in vivo* PDAC phenotypes

To better understand the intercellular crosstalk between stroma and PDAC cells and the influence that such interaction may have on gene expression levels, we made use of the mixed-species co-culture approach allowing for direct RNA extraction from a mixed population of cells followed by an *in silico* separation of the short RNA-seq reads from the mixed libraries. Global transcript level changes for Panc1 cells and mPSCs in heterospheroids versus the respective monospheroids were calculated.

**Fig. 2:**
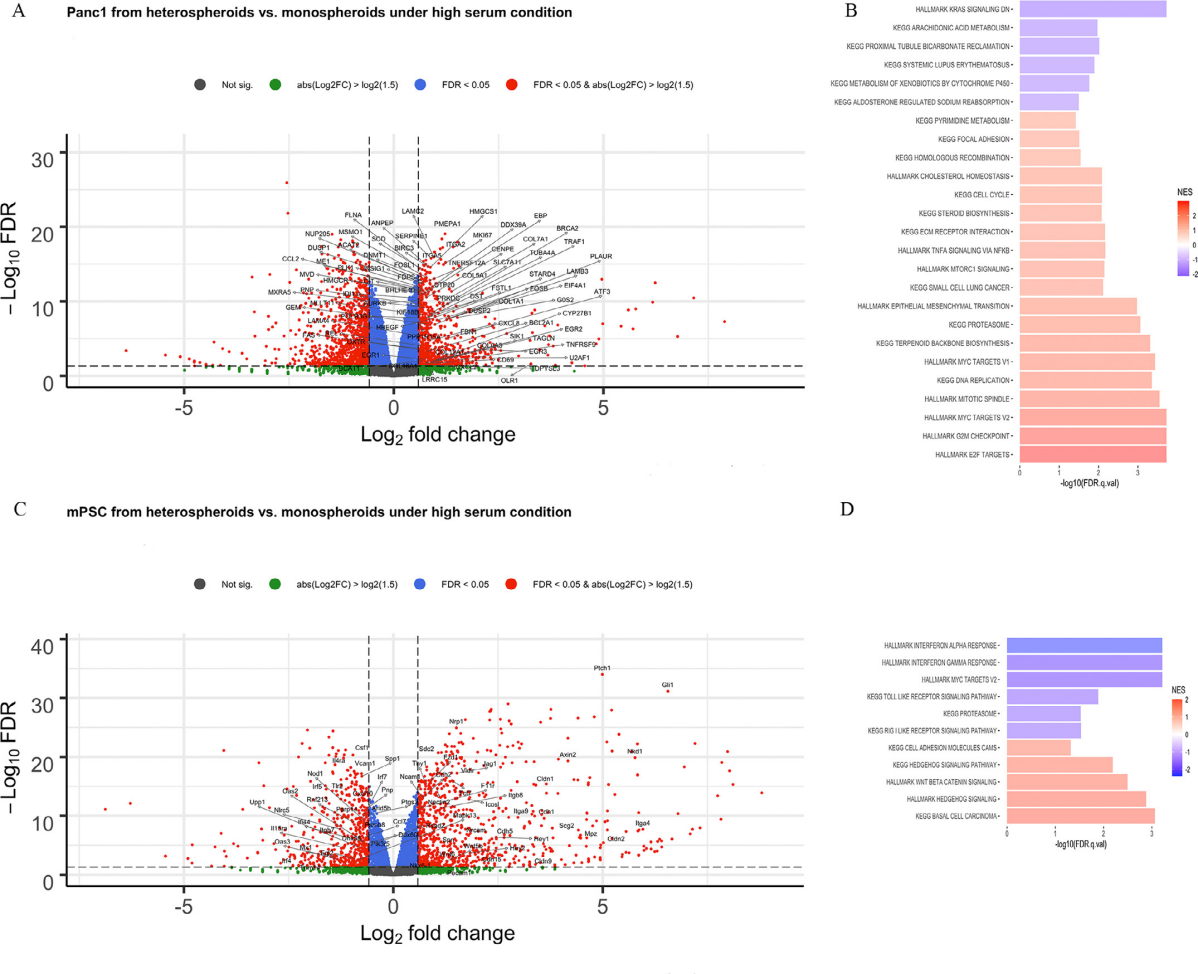
Transcript level changes between heterospheroids and monospheroids under high serum condition (A) Volcano plot showing the genes that are differentially expressed between Panc1 in heterospheroids and monospheroids under high serum condition. (B) Gene set enrichment analysis summarizing the gene sets that were enriched in Panc1 from monospheroids and heterospheroids with FDR *q*-value < 0.05. (C) Volcano plot showing the genes that are differentially expressed between mPSCs in heterospheroids and monospheroids grown in high serum condition. (D) Gene set enrichment analysis summarizing the gene sets that were enriched in mPSCs from monospheroids and heterospheroids with FDR *q*-value < 0.05.

Under high serum condition, 751 DEGs were higher and 1138 were lower in transcript levels for Panc1 cells grown in heterospheroids compared to Panc1 cells grown in monospheroids (Fig. 2A, Supplemental Table S2). The GSEA identified enriched gene sets for genes with higher transcript levels in Panc1 from heterospheroids compared to monospheroids related to cell cycle, DNA replication, epithelial-mesenchymal-transition (EMT) and ECM receptor interaction (Fig. 2B, Supplemental Table S3). We used qRT-PCR to verify that Panc1 from heterospheroids had higher expression of *MKI67, VIM* and *CDH2* compared to monospheroids (Supplemental Figure S2A). In addition, reprogramming of lipid metabolism, including cholesterol homeostasis and steroid biosynthesis, and general signaling pathways including MYC, mammalian target of rapamycin complex 1 (mTORC1) and tumor necrosis factor (TNF) signaling via nuclear factor kappa B (NF-κB) were also enriched in Panc1 from heterospheroids (Fig. 2B, Supplemental Table S3). For mPSCs grown in heterospheroids, 841 DEGs were higher and 547 were lower in transcript levels when compared to mPSCs grown in monospheroids (Fig. 2C, Supplemental Table S4). In addition, the GSEA result showed enriched gene sets for genes with higher transcript levels in mPSCs from heterospheroids compared to monospheroids including hedgehog signaling, Wnt β-catenin signaling and cell adhesion molecules (Fig. 2D, Supplemental Table S5). The mRNA expression of *Gli1,* the effector of hedgehog signaling, in mPSCs from heterospheroids was over 100 times higher than from monospheroids based on qRT-PCR results (Supplemental Figure S2B). On the other hand, an underrepresentation of genes belonging to the gene sets including interferon alpha and -gamma (IFNα, -γ), MYC signaling, Toll like receptor signaling and proteasome was observed in mPSCs from heterospheroids (Fig. 2D, Supplemental Table S5), which corresponds to an enrichment in mPSCs from monospheroids versus heterospheroids.

**Fig. 3:**
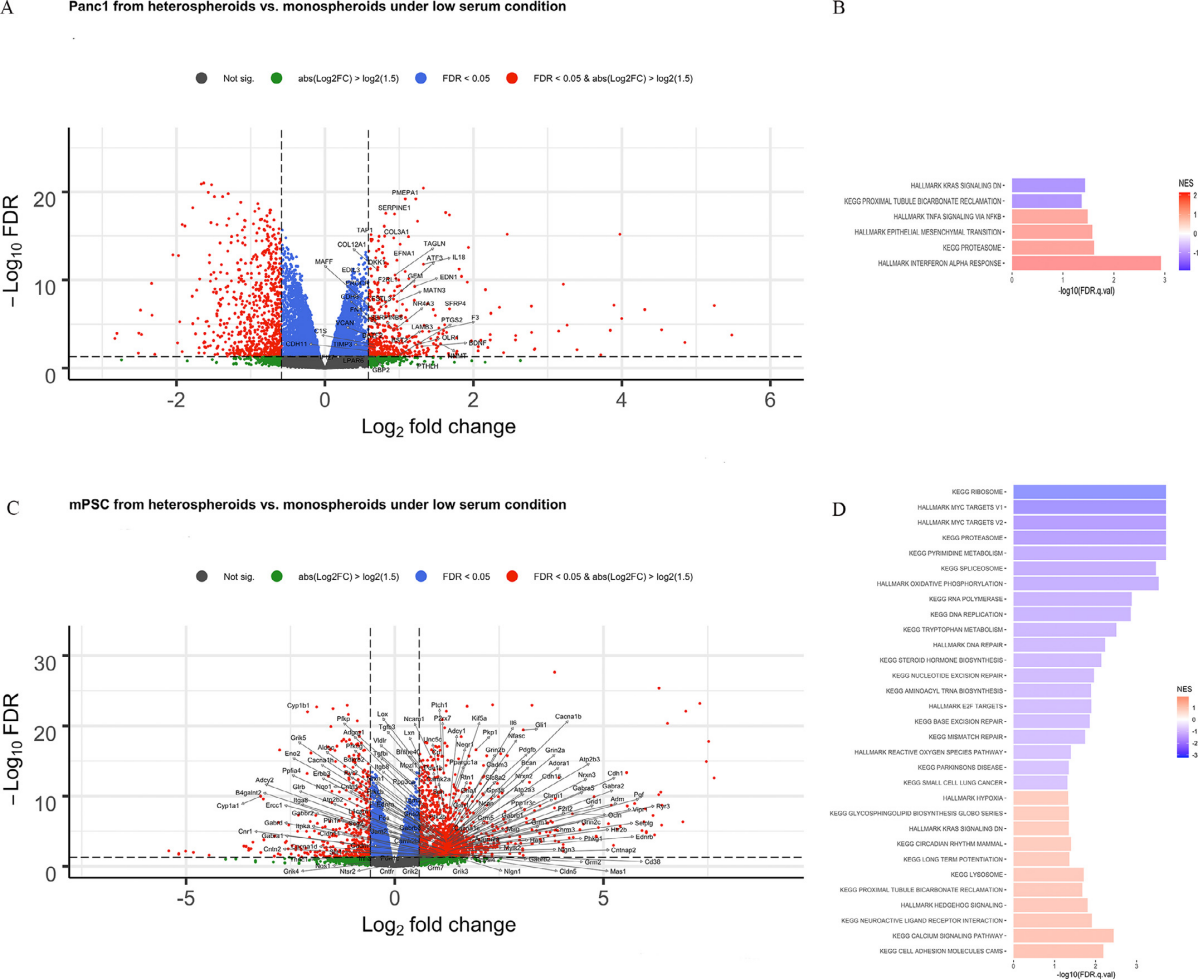
Transcript level changes between heterospheroids and monospheroids under low serum condition (A) Volcano plot depicting the genes that are differentially expressed between Panc1 in heterospheroids and monospheroids under low serum condition. (B) Gene set enrichment analysis summarized the gene sets that were enriched in Panc1 from monospheroids and heterospheroids with FDR q-value < 0.05. (C) Volcano plot showing the genes that are differentially expressed between mPSCs in heterospheroids and monospheroids under low serum condition. (D) Gene set enrichment analysis summarizing the gene sets that were enriched in mPSCs from monospheroids and heterospheroids with FDR *q*-value < 0.05.

Under low serum condition, we found 1160 DEGs in Panc1 cells grown in heterospheroids compared to Panc1 cells grown in monospheroids, of which 441 had higher and 719 had lower transcript levels (Fig. 3A, Supplemental Table S6). The functional gene sets for genes with higher transcript levels in Panc1 from heterospheroids were related to IFNα, proteasome, EMT and TNF signaling via NFκB (Fig. 3B, Supplemental Table S7). We used qRT-PCR to detect the expression of genes involved in EMT and verified that the mRNA expression of *FN1* and *CDH2* was higher in Panc1 from heterospheroids, compared to monospheroids (Supplemental Figure S2C). Independent of serum condition, GSEA results pointed primarily to the functional gene set “genes down-regulated by KRAS activation” for genes with higher transcript levels in Panc1 form monospheroids compared to heterospheroids (Figs. 2B, 3B). For mPSCs grown in heterospheroids, there were 1355 DEGs with higher and 406 with lower transcript levels, compared to mPSCs grown in monospheroids (Fig. 3C, Supplemental Table S8). GSEA results indicated the functional gene sets for genes with higher transcript levels in mPSC form heterospheroids were related to cell adhesion molecules, calcium signaling pathway, neuroactive ligand-receptor interaction, hedgehog signaling, and hypoxia (Fig. 3D, Supplemental Table S9). The expression of *Gli1* in mPSCs from heterospheroids was around nine times higher than from monospheroids (Supplemental Figure S2D). Gene sets of genes with overrepresentation of transcript levels in mPSC from monospheroids included DNA replication and repair, tryptophan metabolism and reactive oxygen species (Fig. 3D, Supplemental Table S9).

### High or low serum conditions had different effects on co-cultured tumor cells and mPSCs

In Panc1 cells, we observed 15 differently expressed protein coding genes were higher and 69 were lower in transcript levels (FDR q-value < 0.05 and absolute fold change > 2) upon co-culture with mPSCs, independent of serum concentration (Supplemental Figure S3A and B). Genes with a higher transcript level and safely above background (RPKM > 5) included *CYR61* (*CCN1*), *CTGF* (*CCN2*) and *PMEPA1* relating to PDAC proliferation and desmoplastic reaction (Supplemental Figure S3C). For mPSCs from hetero- compared to monospheroids, 126 differently expressed protein coding genes were higher and 21 were lower in transcript level (FDR *q*-value < 0.05 and absolute fold change > 2) independent of serum concentration (Supplemental Figure S3D and E). Among the genes with higher transcript expression in mPSCs from heterospheroids were *Acta2*, indicating PSC activation, *Cdh11*, related to cell adhesion, as well as *Ptch1* and *Gli1* involved in the hedgehog pathway (Supplemental Fig. S3F). In addition, we calculated the transcriptional changes of genes with differential regulation upon co-culture between high serum and low serum for Panc1 and mPSCs, respectively (Supplemental Figure S4A and B). The transcript levels of genes that were related to lipid biosynthesis, including *HMGCS1, FASN* and *MVD*, were higher for Panc1 grown in heterospheroids compared to monospheroids under high serum but were lower for Panc1 grown in heterospheroids compared to monospheroids under low serum condition. However, genes involved in interferon signaling, including *IFIT2, IFIT1* and *BST2*, had higher transcript levels in low serum condition but lower transcript levels in high serum condition for Panc1 from heterospheroids compared to monospheroids (Supplemental Fig. S4A). For mPSCs, the transcript levels of genes that were related to the Wnt pathway (*Wnt9a, Axin2, Fzd1, Wisp1* and *Nkd2*), the insulin-like growth factor receptor signaling pathway (*Igf1, Irs1*) and ECM (*Col5a3, Col6a5, Col7a1*) were higher in high serum but lower in low serum in heterospheroids compared monospheroids (Supplemental Figure S4B).

### Targeting cholesterol synthesis led to increased apoptosis in Panc1 upon co-culture with mPSCs

**Fig. 4:**
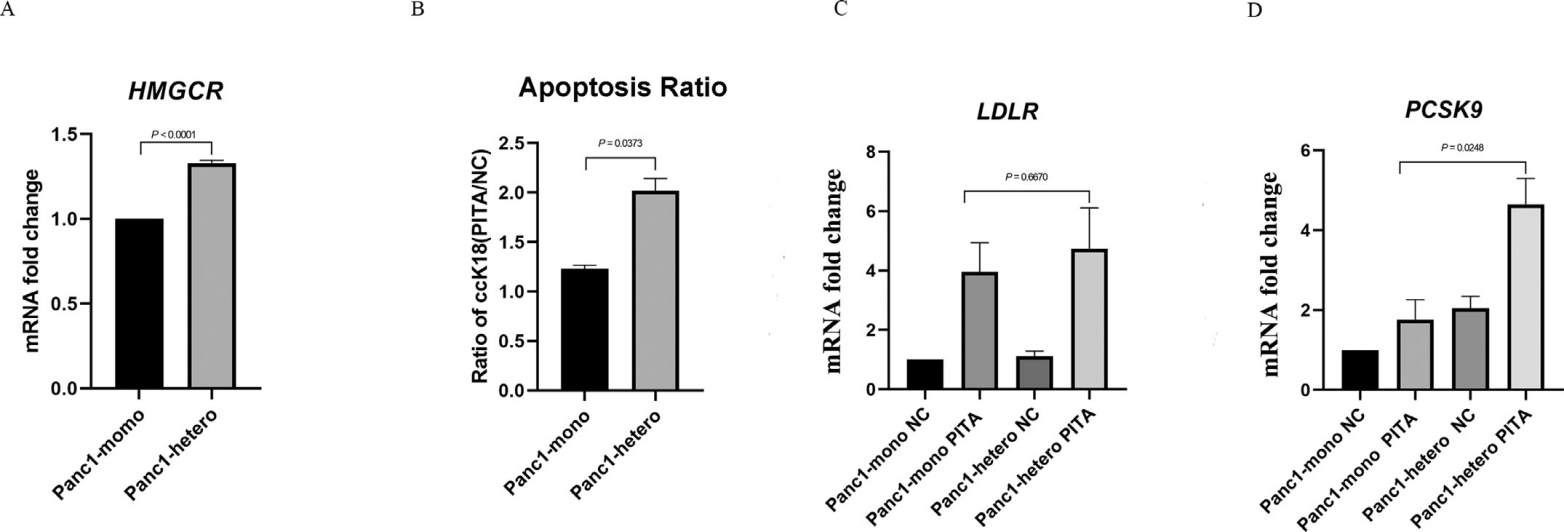
Targeting cholesterol biosynthesis with pitavastatin under high serum condition (A) The mRNA expression of *HMGCR* in Panc1 from monospheroids and heterospheroids was analyzed by qRT-PCR. (B) The ratio of epithelial-specific caspase-cleaved cytokeratin 18 (ccK18) for Panc1 in monospheroids and heterospheroids grown in high serum after 5 µM pitavastatin (PITA) or mock (NC) treatment. (C, D) The expression of *LDLR* and *PCSK9* in Panc1 from monospheroids and heterospheroids grown in high serum and treated with 5 μM pitavastatin. Bars indicate standard error of the mean (SEM).

The genes from the “cholesterol homeostasis” gene set had higher transcript levels in Panc1 from heterospheroids in high serum condition (Fig. 2B). For instance, the expression of 3-hydroxy-3-methylglutaryl coenzyme A reductase (*HMGCR*), the rate-limiting enzyme for cholesterol synthesis, was higher in Panc1 from heterospheroids compared to monospheroids in high serum condition (Fig. 4A). To test whether this identified pathway could be a possible target for PDAC treatment, we incubated Panc1 in monospheroids and heterospheroids under high serum condition with 5 µM pitavastatin, a HMGCR inhibitor, for three days. Panc1 cells in heterospheroids following pitavastatin treatment had a significantly higher apoptosis rate when compared to those grown in monospheroids (Fig. 4B). The mRNA expression of low-density lipoprotein receptor (*LDLR*) in Panc1 from heterospheroids or monospheroids was similar following pitavastatin treatment (Fig. 4C). However, the mRNA expression of proprotein convertase subtilisin/kexin type 9 (*PCSK9*) was significantly elevated in Panc1 from heterospheroids compared to monospheroids following pitavastatin treatment (Fig. 4D).

### Co-culture with pancreatic stellate cells shifted tumor cells to a more aggressive molecular subtype

**Fig. 5:**
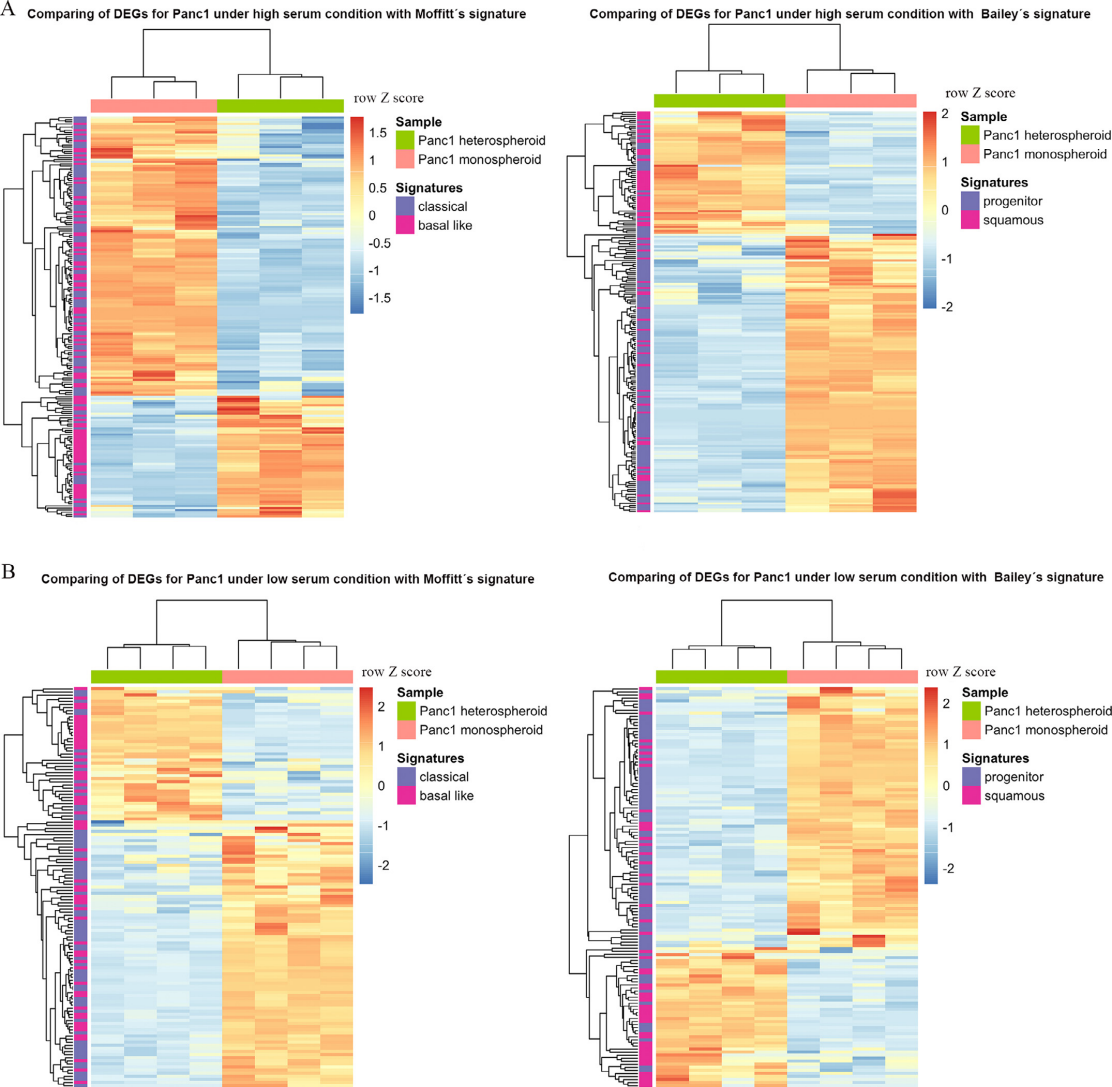
PDAC subtype classification according to previously identified molecular signatures (A) Comparisons of DEGs for Panc1 grown in hetero- vs. monospheroids under high serum condition with the PDAC stratification gene signatures from Moffitt et al. (*P* = 4.13 × 10^−5^) and Bailey et al. (*P* = 5.46 × 10^−8^) [5, 6]. (B) Comparison of the DEGs for Panc1 grown in hetero- vs. monospheroids cultured under low serum condition with the PDAC stratification gene signatures from Moffitt et al. (*P* = 8.45 × 10^−3^) and Bailey et al. (*P* = 1.52 × 10^−5^) [5, 6]. DEGs: differentially expressed genes.

To study whether the transcriptional changes for Panc1 induced by mPSCs co-culture related to previously determined PDAC molecular subtypes, we compared DEGs of Panc1 with the basal-like/classical and squamous/progenitor gene signatures as described by Moffitt et al. [5] and Bailey et al. [6]. Interestingly, a significantly higher proportion of genes from the classical and progenitor signatures had higher transcript levels in Panc1 from monospheroids, while a significantly larger proportion of genes from the basal-like and squamous gene signatures had higher transcript levels in Panc1 from heterospheroids under both, high serum condition (Fig. 5A) and low serum condition (Fig. 5B). Therefore, Panc1 cells in monospheroids appear to have global transcript levels more similar to classical/progenitor, while Panc1 cells in heterospheroids are more reminiscent of basal-like/squamous subtype cells, independent of serum concentration.

### Overlap between significantly changed genes of Panc1 cells co-cultured with mPSCs and pancreatic tumor samples from patients of the TCGA cohort

By analysing the publicly available data of patients with pancreatic adenocarcinoma from the TCGA_PAAD cohort [38] (https://portal.gdc.cancer.gov/projects/TCGA-PAAD) with Moffitt's classification (basal-like vs. classical), we identified 794 DEGs with higher transcript levels in the basal-like subtype patient group and 1086 with higher transcript levels in the classical patient group. For patient groups with Bailey's classification (squamous vs. progenitor), we identified 1107 DEGs with higher transcript levels in the squamous subtype patient group and 1703 DEGs with higher transcript levels in the progenitor subtype patient group. Then we compared the DEGs for Panc1 grown in heterospheroids versus monospheroids with DEGs for patient groups in the TCGA cohort based on Moffitt's and Bailey's classification. Under high serum condition, there was a larger proportion of DEGs with higher transcript levels for Panc1 grown in heterospheroids compared to DEGs with higher transcript levels for Panc1 grown in monospheroids that overlapped with DEGs with higher transcript levels from the basal-like TCGA patient group (6.7% vs. 4.4%, *P* = 0.036, Supplemental Figure S5A) and the squamous TCGA patient group (11.1% vs. 6.3%, *P* = 3.07 × 10^−4^, Supplemental Figure S5B). In addition, there was a bigger proportion of DEGs with higher transcript levels from Panc1 grown in monospheroids compared to that for Panc1 grown in heterospheroids which overlapped with DEGs with higher transcript levels from the classical TCGA patient group (6.3% vs. 1.7%, *P* = 1.12 × 10^−6^, Supplemental Figure S5C) and the progenitor TCGA patient group (11.7% vs. 5.7%, *P* = 3.20 × 10^−5^, Supplemental Figure S5D), respectively. Under low serum condition, the proportion of DEGs with higher transcript levels from Panc1 grown in heterospheroids was bigger than that for Panc1 grown in monospheroids which overlapped with DEGs with higher transcript levels from the basal-like TCGA patient group (7.5% vs. 6.4%, *P* = 0.474, Supplemental Fig. S5E) and the squamous TCGA patient group (12.9% vs. 8.1%, *P* = 8.42 × 10^−3^, Supplemental Figure S5F). However, a larger proportion of DEGs with higher transcript levels from Panc1 grown in monospheroids compared to that for Panc1 grown in heterospheroids overlapped with DEGs with higher transcript levels from the classical TCGA patient group (7.8% vs. 2.7%, *P* = 2.63 × 10^−4^, Supplemental Figure S5G) and the progenitor TCGA patient group (14.9% vs. 7.7%, *P* = 2.82 × 10^−4^, Supplemental Figure S5H), respectively.

### mPSCs changed their CAF subtype depending on co-culture with tumor cells and different serum conditions

**Fig. 6:**
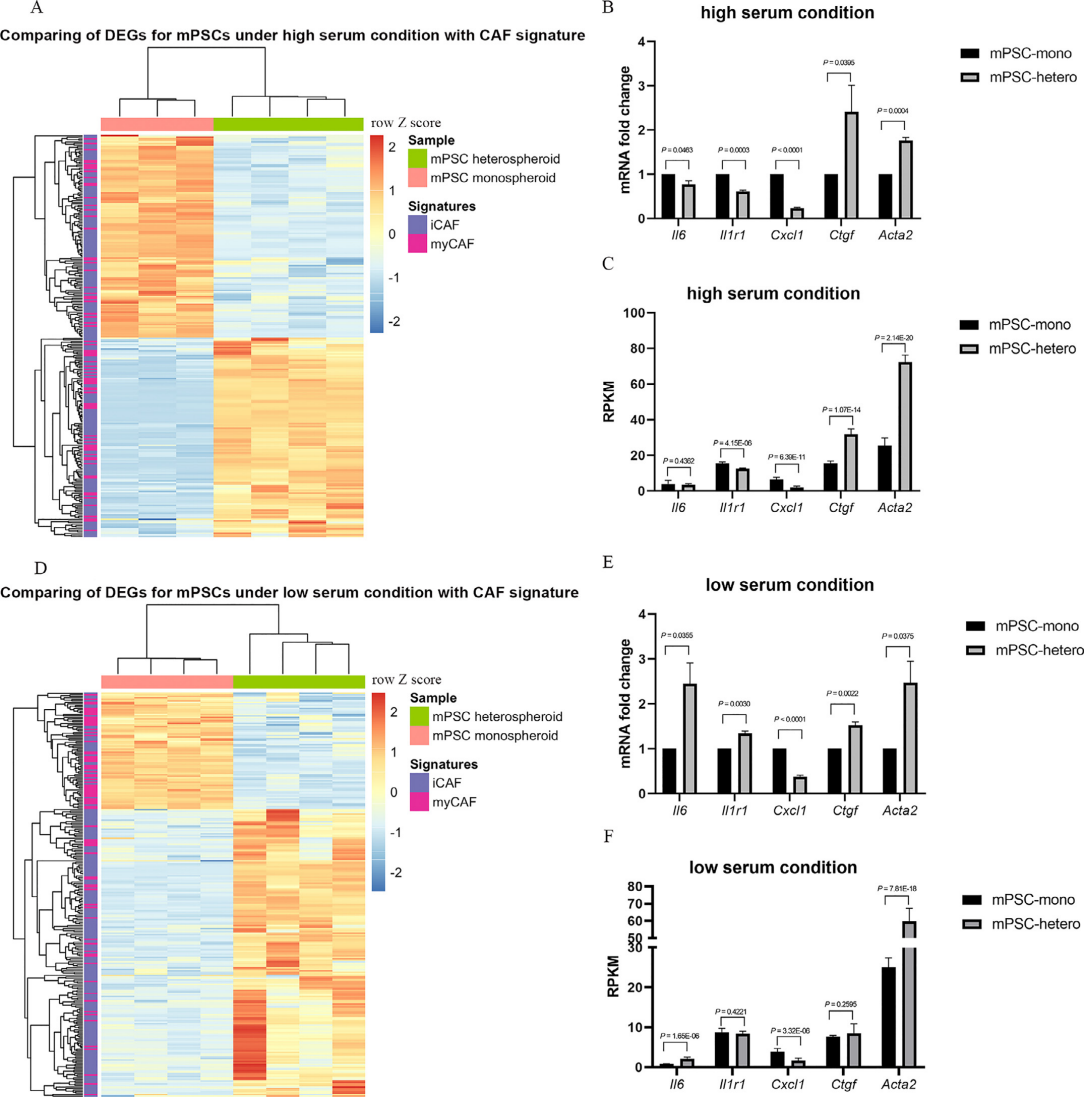
Comparison of genes with significantly changed transcript levels with published CAF signatures (A) Comparison of DEGs for mPSCs grown in hetero- vs. monospheroids under high serum condition with my-/iCAF signatures [11] (*P* = 0.073). (B) qRT-PCR analysis and (C) RPKM value of iCAF and myCAF marker genes in mPSCs from monospheroids and heterospheroids under high serum condition. (D) Comparison of the DEGs for mPSCs grown in hetero- vs. monospheroids under low serum condition with my-/iCAF signatures [11] (*P* = 3.15 × 10^−12^). (E) qRT-PCR analysis and (F) RPKM value of iCAF and myCAF marker genes of mPSCs from monospheroids and heterospheroids under low serum condition. Bars show standard error of the mean (SEM). DEGs: differentially expressed genes; RPKM: Reads Per Kilobase of transcript per Million reads mapped.

Recently, an intratumoral heterogeneity in PDAC has been identified in terms of CAFs, i.e., myCAFs and iCAFs [11]. In order to investigate if similar subtypes of mPSCs might exist in our model, we compared the published signature genes of myCAFs and iCAFs with DEGs of mPSCs in heterospheroids and monospheroids [11]. Under high serum condition, we observed a trend for a larger proportion of genes with higher transcript levels of the myCAF signature from mPSCs grown in heterospheroids, whereas a trend for a larger proportion of genes with higher transcript level from the iCAF signature was detected in mPSCs grown in monospheroids (Fig. 6A). Neverthelesss, qRT-PCR demonstrated that the iCAF marker genes *Cxcl1, Il1r1* and *Il6* were significantly higher expressed in mPSCs from monospheroids, while *Acta2* and *Ctgf* which belong to the myCAF markers had significantly higher expression levels in mPSCs from heterospheroids (Fig. 6B and C). Surprisingly, under low serum condition, we found a statistically significant bigger proportion of iCAF signature genes enriched in mPSCs from heterospheroids, whereas a significantly bigger proportion of myCAF signature genes was enriched in mPSCs from monospheroids (Fig. 6D). These findings were corroborated by qRT-PCR results showing that the iCAF markers *Il6* and *Il1r1* were significantly higher expressed in mPSCs from heterospheroids (Fig. 6E). On the other hand, also the myCAF markers *Cgtf* and *Acta2* were significantly higher expressed and the iCAF marker gene *Cxcl1* was lower expressed in mPSCs from heterospheroids under low serum condition (Fig. 6E). This might reflect either co-existence of two distinct CAF sub-populations in the heterospheroids or that the PSCs/CAFs could exist as a more plastic, mixed phenotype. The trend regarding the expression levels of CAF marker genes from RNA-seq data was consistent with RT-qPCR results except *Il1r1* (Fig. 6F). TGF-β secreted by tumor cells has been demonstrated to promote the myCAF phenotype and counteract iCAF differentiation [41]. In order to test whether the CAF status of mPSCs in heterospheroids under high serum condition could be shifted, we incubated heterospheroids cultured under high serum condition with 5 µM of the TGFBR1/2 kinase inhibitor LY2109761 starting on day 1 after seeding and analyzed the gene expression by qRT-PCR on day 4. The results indicated decreased expression levels of *Ctgf* and *Acta2* in mPSCs from heterospheroids (Supplemental Figure S6), while the expression levels for *Cxcl1* and *Il1r1* increased (Supplemental Figure S6), indicating that inhibition of TGF-β signaling led to a shift of mPSCs from a myCAF to a more iCAF-like phenotype.

### Co-culture with pancreatic stellate cells increased the sensitivity of tumor cells to gemcitabine but not paclitaxel and SN38

**Fig. 7:**
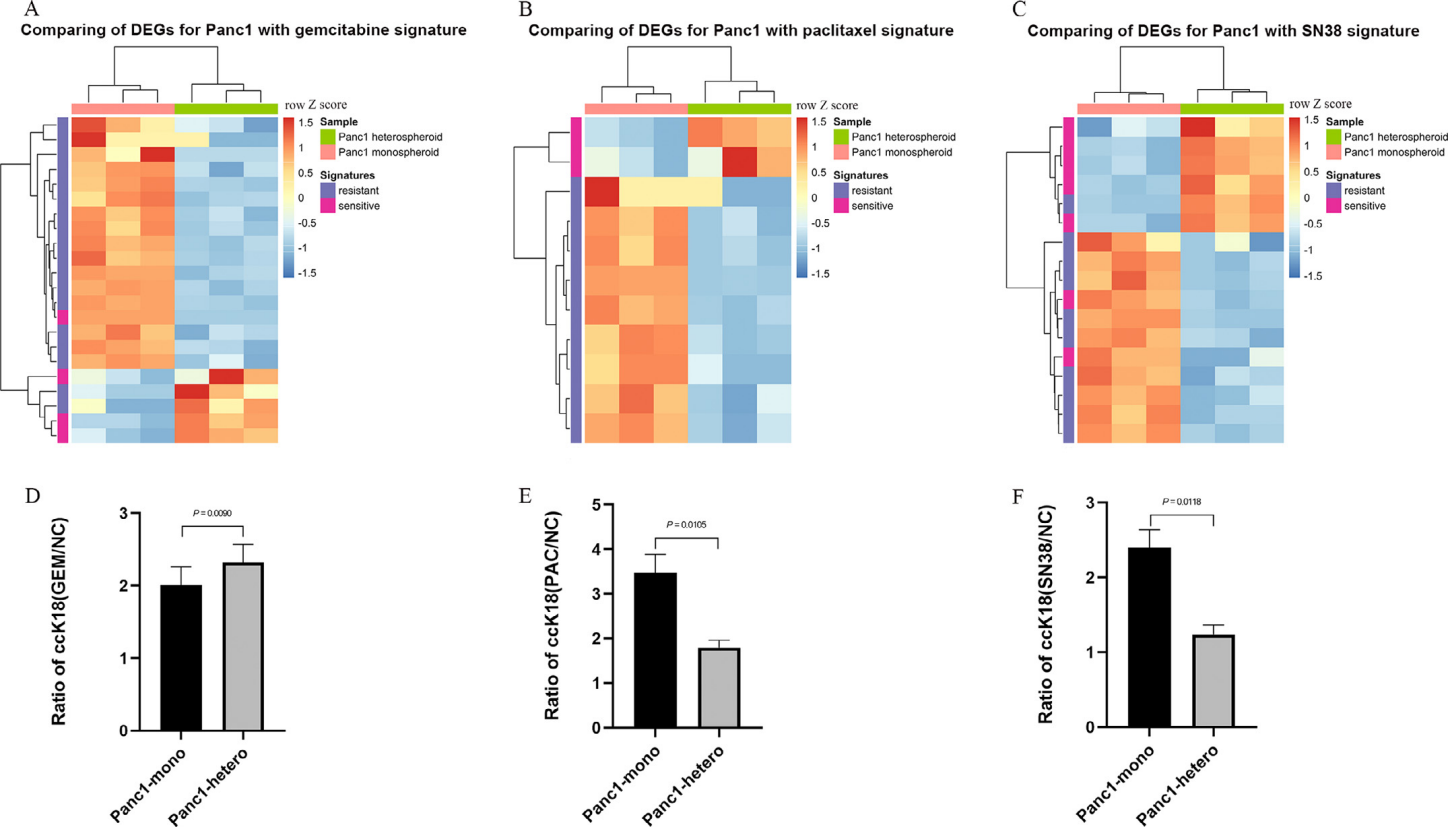
Comparison of genes with significantly changed transcript levels with published pharmacotranscriptomic expression signatures (A–C) Comparison of DEGs for Panc1 grown in hetero- vs. monospheroids under high serum condition with pharmacotranscriptomic expression signatures[36] of (A) gemcitabine (*P* = 0.024), (B) paclitaxel (*P* = 0.018) and (C) SN38 (*P* = 0.035). (D–F) The ratio of epithelial specific caspase-cleaved cytokeratin 18 (ccK18) for Panc1 from monospheroids and heterospheroids after 50 µM gemcitabine (D), 1 μM paclitaxel (E) and 1 μM SN38 (F) treatment on day 1 and analyzed on day 4 under high serum condition. Bars show standard error of the mean (SEM). GEM: gemcitabine; PAC: paclitaxel; DEGs, differentially expressed genes.

Chemotherapy is the major treatment for most PDAC patients. Gemcitabine plus nanoparticle albumin-bound paclitaxel and FOLFIRINOX (a combination of folinic acid/leucovorin, 5-FU, irinotecan and oxaliplatin) are new first-line therapies for metastatic PDAC patients [42,43]. Recently, pharmacotranscriptomic expression signatures derived from PDAC patients’ organoids have been proposed to predict responses to several commonly used chemotherapeutics [36]. In order to investigate whether these pharmacotranscriptomic expression signatures can predict changes in treatment response of Panc1 cells upon coculture with mPSCs, we compared the DEGs of Panc1 grown under high serum condition with the published pharmacotranscriptomic expression signatures for gemcitabine (GEM), paclitaxel (PAC) and SN38 [36]. A larger proportion of genes predicting “sensitive to drug” had higher expression levels in Panc1 from heterospheroids under high serum condition for all three drugs (Fig. 7A–C). Since it is technically difficult to analyze the treatment effect on the apoptosis level of only the Panc1 cells in the heterospheroids, we used the M30 Apoptosense® CK18 kit, which detects the epithelial-specific soluble caspase-cleaved cytokeratin 18 (ccK18), to determine apoptosis specifically in the cancer cells. A significantly higher apoptosis rate was identified for Panc1 from heterospheroids compared to monospheroids after 50 µM gemcitabine treatment for 3 days (Fig. 7D). A heatmap shows the transcript levels of gemcitabine importer genes (*SLC29A1, SLC29A2*) and metabolizing enzymes (*DCK, CMPK1* and *NME1*) (Supplemental Figure S7A). Most of these genes had higher transcript levels in Panc1 from heterospheroids compared to monospheroids. We verified these observations by analyzing the expression of *SLC29A1* (major gemcitabine transporter) and *DCK* (rate-limiting activating enzyme) by qRT-PCR and found Panc1 had significantly higher expression of these two genes upon co-culture with mPSCs (Supplemental Figure S7B and C). In contrast to gemcitabine, treatment with 1 µM paclitaxel (Fig. 7E), or 1 µM SN38 (Fig. 7F) for 3 days resulted in significantly higher apoptosis rates for Panc1 cells from monospheroids compared to heterospheroids, which was not predicted by comparison to the respective pharmacotranscriptomic signatures.

## Discussion

Desmoplasia represents a morphological and functional hallmark in pancreatic cancer. Much is known about the tumor cells as driver of the fibrogenic response by e.g., secretion of TGF-β [8]. However, studying the deeper interrelationships and crosstalk between the different cellular compartments of PDAC has been difficult, partly due to lack of suitable models. Although genetically engineered mouse models and xenograft models exist that more or less reflect tumor microenvironment, they are labour-intensive, expensive and time-consuming [44]. Here, we present and characterize an advanced *in vitro* 3D co-culture system, which can be employed to study interactions between tumor cells and PSCs/CAFs under reproducible and controllable conditions suitable for interventions, while being monitorable through measurements of changes in gene expression levels in either of the involved cell types.

### The hallmarks of PDAC are reflected in the heterospecies heterospheroid model under different culture conditions

Under high serum condition, genes reflecting the main characteristics of PDAC, including proliferation, EMT, ECM receptor interaction and cholesterol homeostasis, were found to have increased transcript levels in Panc1 cells when co-cultured with mPSCs (Fig. 2B and Supplemental Figure S2A). At the same time mPSCs got activated, indicated by elevated expression of *Acta2* (Fig. 6B and C). Increased proliferation and EMT of pancreatic cancer cells and PSC activation have also been observed in a microfluidic 3D-culture system [45]. Recently, a scRNA-seq study demonstrated that PDAC cells co-cultured with CAFs resulted in elevated proliferation and EMT compared to PDAC cell monocultures [46]. Especially, some of the tumor cells were found displaying both proliferation and EMT phenotypes simultaneously. The molecular mechanism behind this observation was the activation of MAPK and STAT3 signaling pathways in tumor cells induced by CAF-secreted TGF-β1 [46]. We found the hedgehog pathway enriched in mPSCs from heterospheroids, with around 32-fold higher expression of *Ptch1* and 100-fold higher expression of *Gli1* (Fig. 2C and Supplemental Figure S2B). The role of sonic hedgehog (Shh) signaling in PDAC is controversial. It has been found supporting the desmoplastic reaction in PDAC [47], while prolonged or constitutive inhibition of Shh signaling in genetically engineered mouse models led to undifferentiated, more aggressive PDAC cells and decreased survival [48]. These unexpected observations paved the way for the idea that CAFs with tumor suppressive property might exist in PDAC [49]. Therefore, stroma targeting treatment strategies need to be developed by taking into account the effects of CAFs and as well as other stromal cell types.

The nutrient poor environment is one of the main characteristics of PDAC, which is caused by the excessive ECM formation and paucity of a functional vasculature inside the tumor, contributing to the aggressive phenotype and chemoresistance [50]. We mimicked the poor nutrient condition in our advanced *in vitro* model by culturing cells under low serum condition and found inflammation-related pathways such as IFNα and TNF enriched in Panc1 from heterospheroids compared to monospheroids (Fig. 3B). These findings are well in line with a previous scRNA-seq analysis of primary PDAC tumor tissue [51]. Recently, another scRNA-seq analysis indicated that the inflammatory-related pathways including IFNα and -γ signaling and TNF signaling were enriched in parental tumor tissue, whereas cell cycle related pathways were enriched in organoids [52]. Therefore, we speculate that the low serum condition might more closely mimic the PDAC *in vivo* environment, especially for avascular tumor microregions. Furthermore, the most widely used *in vitro* cell culture condition, supplementation with high concentrations of fetal calf serum, might constitute a bias towards a mainly proliferative response.

### Co-culture of PDAC cells and murine pancreatic stellate cells revealed a switch in tumor and CAF phenotype

Research during the last 20 years has clearly demonstrated that cancer and stromal cells in PDAC and other cancers influence each other strongly [[4], [5], [6],^11^]. In a reductionist approach, this was much neglected in preclinical research until recently. Here, we demonstrated that tumor cells co-cultured with PSCs shifted towards a more squamous/aggressive phenotype and simultaneously educated their local microenvironment (Fig. 5A, B and 6A, D). Furthermore, we validated such tumor subtype switch of Panc1 cells by comparing their transcriptomes to that of patient samples from the TCGA_PAAD cohort [38]. We found that Panc1 cells grown in heterospheroids behaved more similar to tumors/patients of the basal-like/squamous subtype, while Panc1 cells in monospheroids were closer to tumors of the classical/progenitor subtype, independent of serum concentration (Supplemental Fig. S5). The molecular basis for progenitor/classical and squamous/basal-like differentiation is less known. It has been proposed that TP63-mediated enhancer reprogramming drives squamous subtype differentiation in PDAC [53]. However, TP63 has not been found to be expressed in Panc1 cells [54], which was in line with our RNA-seq results (the observed expression level for *TP63* was less than 0.2 RPKM). Hence, there must be a TP63-independent mechanism driving Panc1 to the more aggressive subtype in our model. MYC amplification has been found associated with adenosquamous histology in PDAC [55]. Bailey et al. demonstrated that MYC pathway activation is involved in the squamous subtype [6]. Interestingly, we found gene sets representing MYC targets enriched in Panc1 cells when grown in heterospheroids (Fig. 2B), indicating that MYC might be a driver of this shift towards a more aggressive cancer subtype.

Öhlund et al. [11] were the first to distinguish two subtypes of CAFs with different spatial location and functions, while a possible molecular mechanism to explain the different subtypes was identified by Biffi et al. [41]. We found that the subtype of the CAFs was not only affected by co-culture with tumor cells, but also by the culture conditions themselves. Under low serum condition, the inflammation-related pathway genes enriched in Panc1 from heterospheroids might provide an inductive stimulus for the iCAF phenotype of the co-cultured mPSCs, while Panc1 from heterospheroids cultured in high serum condition might favour mainly a myofibroblast supporting environment, due to for instance high content of platelet-derived growth factor and TGFβ in FBS.

### PSCs/CAFs influenced the sensitivity of tumor cells towards therapeutic approaches in the mixed species heterospheroid model

The poor response of PDAC patients to drug treatment depends on tumor cell-autonomous characteristics and interactions with surrounding cells of the tumor microenvironment. We found that the presence of mPSCs significantly influenced the therapeutic response of Panc1 cells in our model. Based on a previous observation that CAFs could act as a sink for gemcitabine [56], one may have expected that the presence of mPSCs has a protective effect on Panc1 cells in the heterospheroids. However, we observed a higher apoptosis rate of Panc1 cells upon co-culture with mPSCs, which was supported by comparison of our RNA expression data to the recently published pharmacotranscriptomic gemcitabine sensitivity signature [36] (Fig. 7A, D). These results are also in line with previous studies by Collision et al. [4] and Chan-Seng-Yue et al. [57], who found that quasi-mesenchymal and basal-like PDACs were more sensitive to gemcitabine. The reason for the increased sensitivity towards gemcitabine might be the higher expression of gemcitabine importers (*SLC29A1, SLC29A2*) and prodrug activating enzymes (*DCK*) in Panc1 upon co-culture with mPSCs (Supplemental Figure S7A, B and C) [58]. In contrary to the predictions deduced from the paclitaxel and SN38 pharmacotranscriptomic signature comparison to Panc1 DEGs (Fig. 7B and C), our results of epithelial cell specific apoptosis assays indicated that Panc1 cells were more sensitive to paclitaxel and SN38 in monospheroids (Fig. 7E and F). This means that the presence of stromal cells in the form of mPSCs seemed to have a protective effect on the co-cultured Panc1 cells for these drugs. Our results also indicate that the pharmacotranscriptomic signatures derived from pure epithelial organoid cultures might not be translatable one to one on stroma-containing culture approaches. Paclitaxel is a microtubule-stabilizing agent, and a well known mechanism of paclitaxel resistance is overexpression of the ATP-binding cassette (ABC) transporter ABCB1, which leads to efflux of paclitaxel [59]. However, the transcript level of *ABCB1* was extremely low in all types of Panc1 spheroids. In addition, another ABC transporter ABCG2 has been found to be related to SN38 resistance in colon cancer and breast cancer cells [60,61]. However, we found no statistical difference in the expression of *ABCG2* between Panc1 from monospheroids and heterospheroids. Therefore, there might exist other mechanisms contributing to paclitaxel and SN38 resistance in our heterospecies heterospheroids, e.g., the more compacted structure (Supplemental Figure S1C) that could hamper drug penetration.

### Statins might be promising candidates to treat highly proliferative PDAC cells with cholesterol dependency

In order to test the importance of the upregulated pathways in cancer cells upon co-culture, we chose to target cholesterol synthesis by pitavastatin, a HMG-CoA reductase inhibitor. Statins have been shown to lower the risk of pancreatic cancer development, but little is known about the therapeutic effect of statins on established PDAC [62]. Our results demonstrated that pitavastatin treatment induced apoptosis on Panc1 cells, and co-culture with mPSCs increased this response (Fig. 4B). The reason might be the strongly elevated expression of *PCSK9* in Panc1 from heterospheroids following pitavastatin treatment (Fig. 4D). PCSK9 has the role of decreasing low-density lipoprotein cholesterol uptake by binding to extracellular LDLR, leading to LDLR internalization and intracellular degradation [63]. Our findings suggest statins as promising candidates for the treatment of highly proliferative PDAC with cholesterol dependency, both through inhibiting biosynthesis and impairing uptake.

### Assessment of advantages of the heterospecies spheroid model

Three-dimensional co-cultivation of different cell types has recently been widely used to narrow the gap between traditional monolayer culture and animal models [64]. The crosstalk between stroma and cancer cells has been investigated through indirect co-culture methods that neglect physical interaction [65] or direct co-culture that is hard to track back to the individual cell type. The advantage of the model described in the present study is that it allowed us to monitor the cells without any manipulation (e.g., enzymatic and or physical dissociation) of the spheroid itself prior to harvesting the RNA. The feasibility of the heterospecies spheroid PDAC model has been demonstrated by us [19] and others who studied co-culture of MIA PaCa-2 with mouse embryonic fibroblast [66]. Another advantage is that our spheroid model is scaffold-free, precluding biomaterials that might interfere with therapeutic agents [16,19]. Despite the obvious advantages of the mixed-species system revealed by our study, there are also limitations. Some signaling molecules may be species-specific, therefore the crosstalk between human tumor and mouse stroma cells might lack certain aspects [67]. However, our previous study confirmed that mouse PSCs and human PSCs behaved very similar when co-cultured with Panc1 [19], which could partly reduce the concern of species difference between mouse and human PSC.

## Conclusion

We established and characterized an advanced heterospecies 3D co-culture model, which recapitulated key features of *in vivo* PDAC for both tumor cells and PSCs/CAFs. Co-culture shifted the cancer cells towards a more aggressive molecular subtype, while at the same time not only activating mPSCs, but also influencing their cancer-related phenotype (iCAF/myCAF). In addition, co-cultured mPSCs affected the drug sensitivity of cancer cells significantly, making stromal cells a non-neglectable parameter when searching for new therapeutic strategies and in drug testing. Furthermore, this culture approach is also most suitable for elucidating the effects of CRISPR-Cas mediated gene manipulation, setting the stage for studying the mechanism of microenvironmental influence on PDAC as well as high-throughput drug screening.

## Funding

This study was supported with financial grants by Vetenskapsrådet (grant number: K2013-67 × 22322–01-3), RaHFo (grant numbers: 111252, 131163), EPC-TM-Net (EU grant number: 256974) and PRECODE (EU grant number: 861196) to ML, and CancerFonden (grant numbers: CAN2013/780, CAN2017/615, 20 1356 PjF 01 H) to RH and CancerFonden (grant number: CAN2018/624) to AW as well as China Scholarship Council (scholarship number: 201700260279) to XL.

## Credit authorship contribution statement

**Xinyuan Liu**: Software, Data Curation, Investigation, Writing - Original Draft preparation, Visualization, Funding acquisition. **Beate Gündel**: Investigation, Writing - Review & Editing. **Xidan Li**: Data Curation. **Jianping Liu**: Investigation. **Anthony Wright**: Methodology, Funding acquisition. **Matthias Löhr**: Conceptualization, Resources, Writing - Review & Editing, Supervision, Funding acquisition. **Gustav Arvidsson**: Methodology, Software, Data Curation. **Rainer Heuchel**: Conceptualization, Resources, Writing - Review & Editing, Supervision, Funding acquisition.

## Declaration of Competing Interests

The authors declare that they have no known competing financial interests or personal relationships that could have appeared to influence the work reported in this paper.

## Appendix Supplementary materials

Image, application 1

Image, application 2

Image, application 3

Image, application 4

Image, application 5

Image, application 1

Image, application 7

Image, application 8

Image, application 9

Image, application 10
