# Supplementary material for: 3D heterospecies spheroids of pancreatic stroma and cancer cells demonstrate key phenotypes of pancreatic ductal adenocarcinoma
Source: Transl Oncol. 2021 May 1;14(7):101107. doi: 10.1016/j.tranon.2021.101107 (PMC8111319; doi:10.1016/j.tranon.2021.101107)
Supplement: Supplementary file 1 [file mmc1.docx]

| Primer Pair | Direction | Sequence |
| --- | --- | --- |
| RPL13A | forward | GTCGTACGCTGTGAAGGCA |
|  | reverse | TTTCTTGTCGTAGGGCGGTG |
| PCSK9 | forward | CCAAGATCCTGCATGTCTTCC |
|  | reverse | AACTTCAAGGCCAGCTCCAG |
| LDLR | forward | CAGATATCATCAACGAAGC |
|  | reverse | CCTCTCACACCAGTTCACTCC |
| HMGCR | forward | CTGTCATTCCAGCCAAGGTT |
|  | reverse | GTCCACAGGCAATGTAGATG |
| MKI67 | forward | CTTTGGGTGCGACTTGACG |
|  | reverse | ACCCCGCTCCTTTTGATAGTA |
| CDH2 | forward | CTGCCATGACGTTTTATGGT |
|  | reverse | GGTTTGACCACGGTGACTAA |
| VIM | forward | GAACCAATGAGTCCCTGGAA |
|  | reverse | AGGTGGCAATCTCAATGTCA |
| FN1 | forward | GCAGCCTGCATCTGAGTACA |
|  | reverse | GGTGGAATAGAGCTCCCAGG |
| SLC29A1 | forward | GAGCACGATGCCTGGTTCAT |
|  | reverse | CAATGTCCCCACTGTCCGTC |
| DCK | forward | GAGGTGCCTATCTTAACACTGGA |
|  | reverse | AAAAGCCAACCTGCTGAGGA |
| Rpl13a | forward | GGCCAAGATGCACTATCGGA |
|  | reverse | GTGCGCTGTCAGCTCTCTAA |
| Acta2 | forward | GTCCCAGACATCAGGGAGTAA |
|  | reverse | TCGGATACTTCAGCGTCAGGA |
| Ctgf | forward | AAAGCAGCTGCAAATACCAA |
|  | reverse | TGTCTTCCAGTCGGTAGGCA |
| Il1r1 | forward | GGAATGTGGCTGAAGAGCAC |
|  | reverse | CCCGTGACGTTGCAGATCAG |
| Cxcl1 | forward | ACCTCAAGAACATCCAGAGCT |
|  | reverse | AGGTGCCATCAGAGCAGTCT |
| Il6 | forward | AGTTGCCTTCTTGGGACTGA |
|  | reverse | TCAGAATTGCCATTGCACAA |
| Gli1 | forward | CTGCGTGGTAGAGGGAACTC |
|  | reverse | CCTGGGACCCTGACATAAAG |
