# Supplementary material for: 3D heterospecies spheroids of pancreatic stroma and cancer cells demonstrate key phenotypes of pancreatic ductal adenocarcinoma
Source: Transl Oncol. 2021 May 1;14(7):101107. doi: 10.1016/j.tranon.2021.101107 (PMC8111319; doi:10.1016/j.tranon.2021.101107)
Supplement: Supplementary file 10 [file mmc10.docx]

**Supplementary Information**

3D heterospecies spheroids of pancreatic stroma and cancer cells demonstrate key phenotypes of pancreatic ductal adenocarcinoma

Xinyuan Liu^a^, Beate Gündel^a^, Xidan Li^b^, Jianping Liu^b^, Anthony Wright^c^, Matthias Löhr^a,†^, Gustav Arvidsson^c,†^ and Rainer Heuchel^a,†,⁎^ rainer.heuchel@ki.se

^a^Pancreas Cancer Research Lab, Department of Clinical Science, Intervention and Technology, (CLINTEC), Karolinska Institutet, Huddinge SE 141 86, Sweden

^b^Department of Medicine, Karolinska Institutet, Huddinge SE 141 86, Sweden

^c^Division of Biomolecular and Cellular Medicine, Department of Laboratory Medicine, Karolinska Institutet, Huddinge SE 141 86, Sweden

⁎Corresponding author

†equal contribution

**Supplemental Figures**

**Supplemental Figure S1**


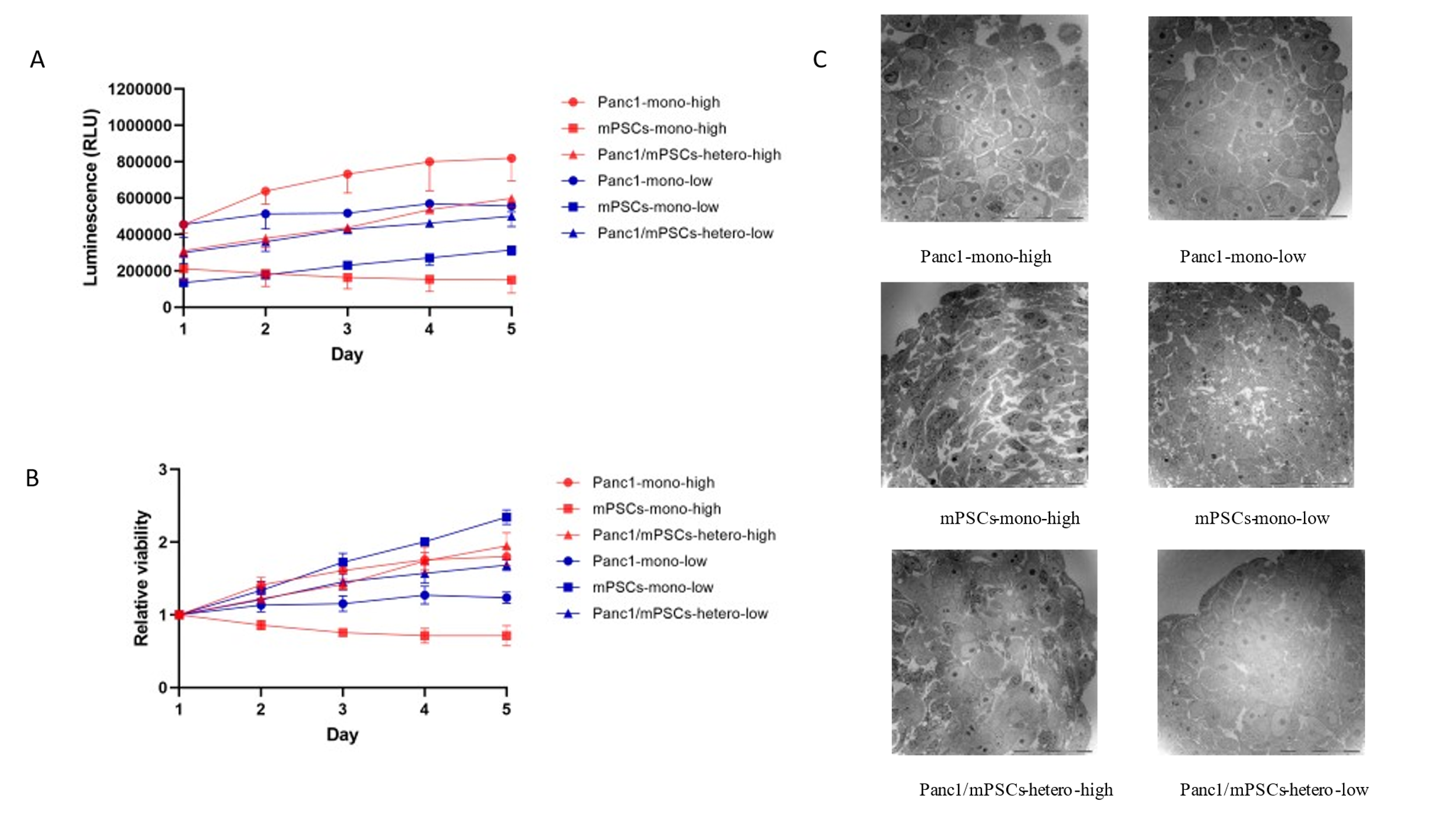


**Supplemental Figure S1. Growth curves for Panc1 and mPSCs from monospheroids and heterospheroids**

(A) Viability of Panc1, mPSCs from monospheroids (2500 cells) and heterospheroids (starting ratio: 500 Panc1 cells plus 2000 mPSCs under high serum and 1250 Panc1 cells plus 1250 mPSCs under low serum condition) from day 1 to day 5. (B) Relative viability of Panc1, mPSCs from monospheroids and heterospheroids defined by normalization to values at day 1. (C) Transmission electron microscopy of Panc1 and mPSCs monospheroids and heterospheroids sections from day 5 under high serum and low serum condition. Scale bars correspond to 50μm.

**Supplemental Figure S2**


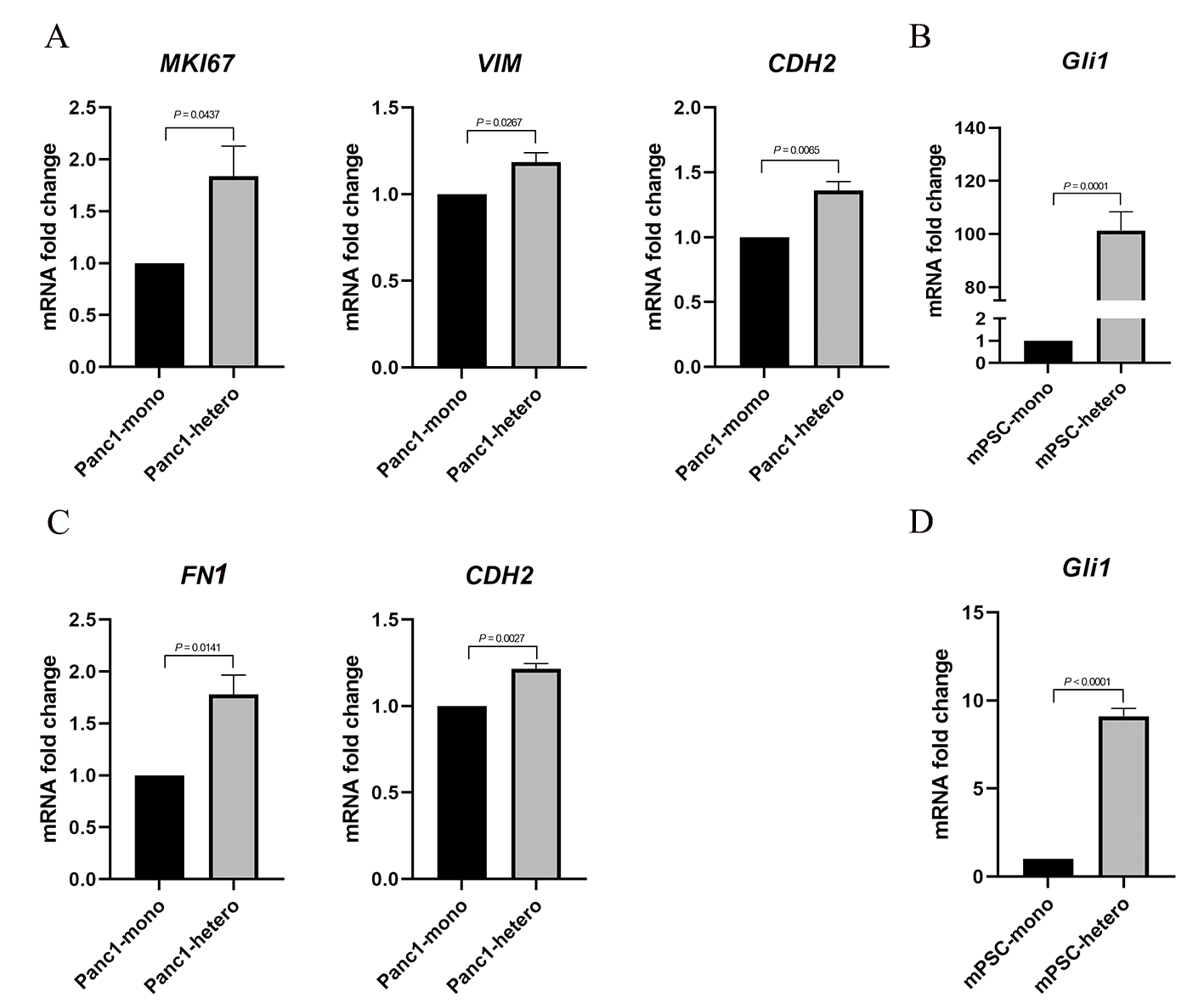


**Supplemental Figure S2. Verification of mRNA expression for marker genes related to proliferation, EMT and hedgehog signaling by qRT-PCR.**

(A) mRNA expression of *MKI67*, *VIM* and *CDH2* in Panc1 from monospheroids and heterospheroids under high serum condition. (B) mRNA expression of *Gli1* in mPSCs from monospheroids and heterospheroids under high serum condition. (C) mRNA expression of *FN1* and *CDH2* in Panc1 from monospheroids and heterospheroids under low serum condition. (D) mRNA expression of *Gli1* in mPSCs from monospheroids and heterospheroids under low serum condition. Bars show standard error of the mean (SEM).

**Supplemental Figure S3**

**
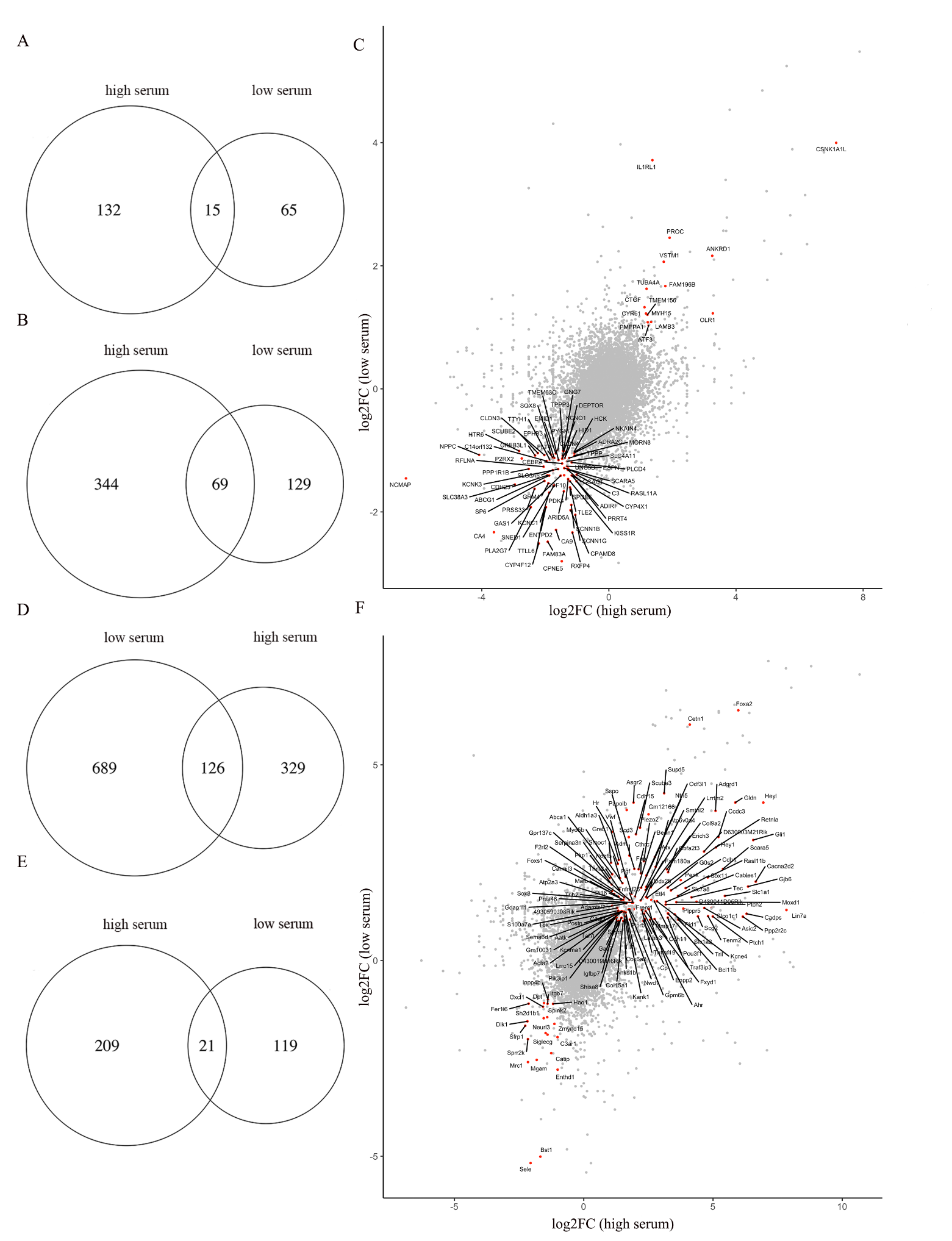
**

**Supplemental Figure S3. Similar transcriptional expression changes due to co-culture under high and low serum conditions.**

(A, B) Venn plots showing the overlapping differently expressed protein coding genes of Panc1 with fold change > 2 higher (A) and lower (B) in transcript level upon co-culture with mPSCs independent of serum concentration. (C) Dot plot showing the overlapping genes from A and B with log_2_ fold change in high serum and low serum conditions. (D, E) Venn plots showing the overlapping differently expressed protein coding genes of mPSCs with fold change > 2 higher (D) and lower (E) in transcript level upon co-culture with Panc1 independent of serum concentration. (F) Dot plot showing the overlapping genes from D and E with log_2_ fold change under high and low serum conditions. log2FC: log_2_ fold change.

**Supplemental Figure S4**

**
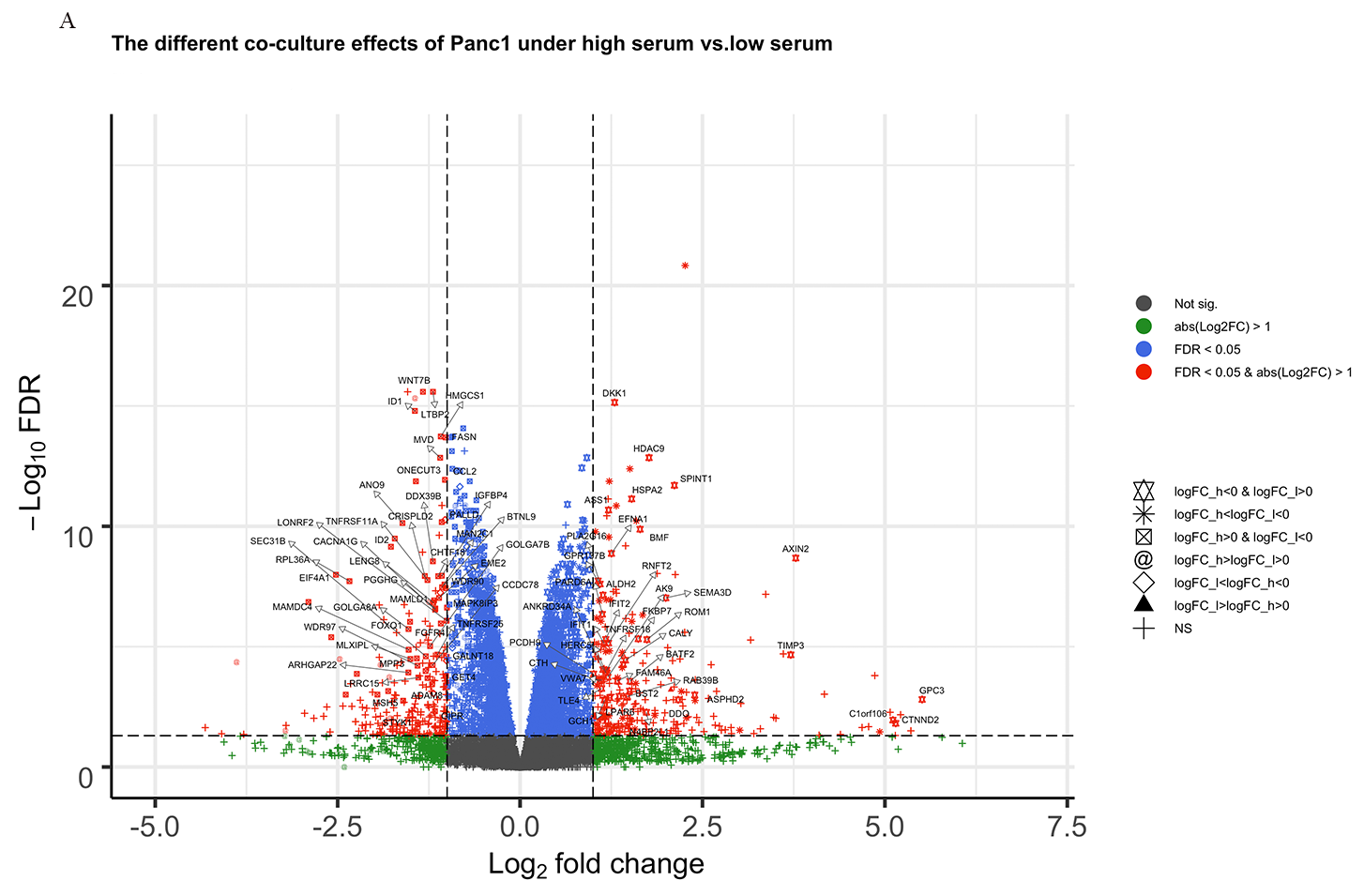
**

For A and B

**
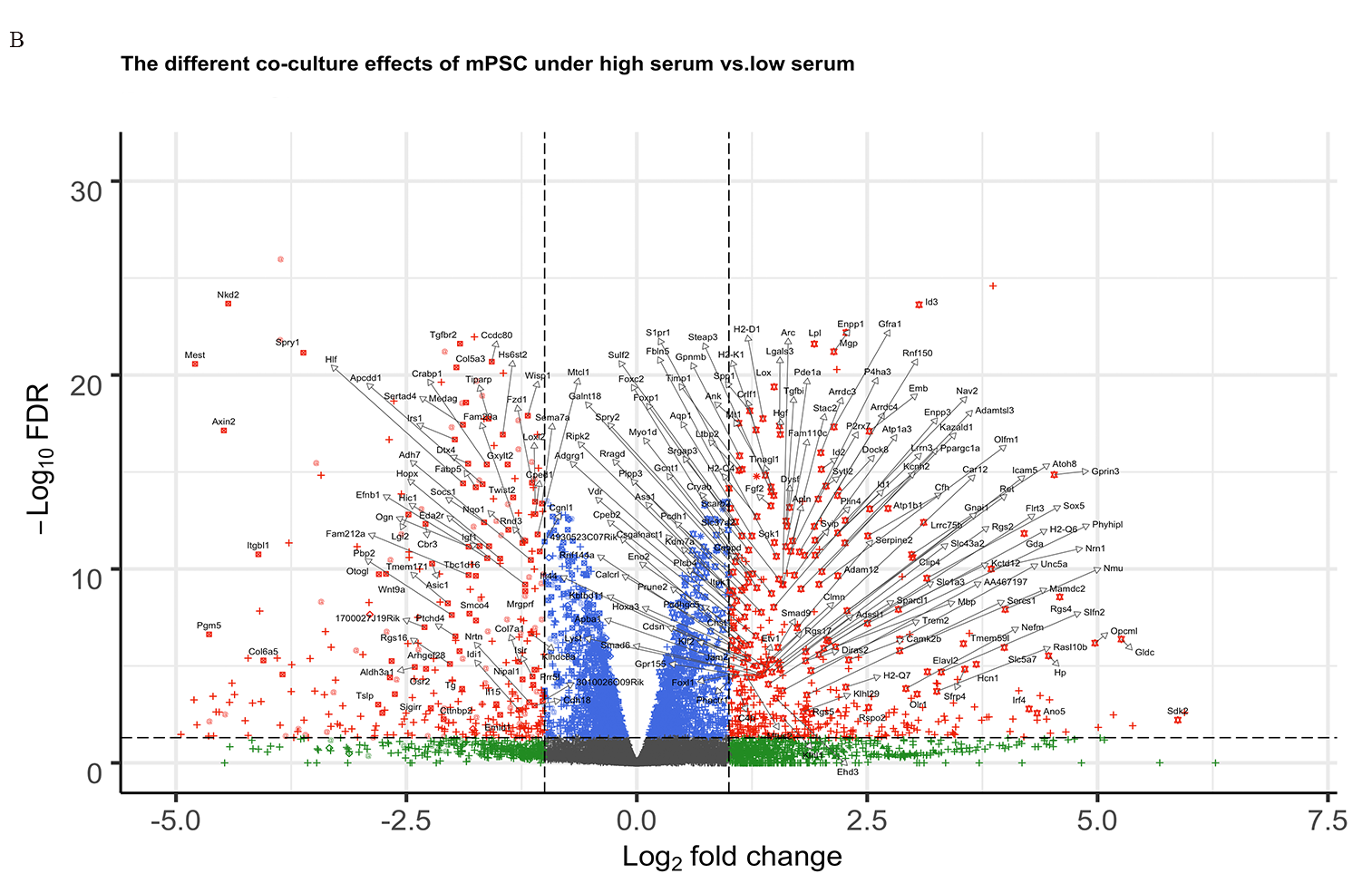
**

**Supplemental Figure S4. The distinctions of the cocultivation effects in transcriptional changes between high and low serum conditions.**

(A) Volcano plot showing the comparison of genes in Panc1 that were differentially expressed between heterospheroids versus monospheroids under high serum condition and heterospheroids versus monospheroids under low serum condition. (B) Volcano plot showing the comparison of genes in mPSCs that were differentially expressed between heterospheroids versus monospheroids under high serum condition and heterospheroids versus monospheroids under low serum condition. The protein coding genes differently expressed upon co-culture between these two conditions (higher expression under high serum condition but lower expression under low serum condition or lower expression under high serum condition but higher expression under low serum condition) with absolute log_2_ fold change more than 1 and FDR < 0.05 have been labelled. LogFC_l: log_2_ fold change between heterospheroids versus monospheroids under low serum condition; LogFC_h: log_2_ fold change between heterospheroids versus monospheroids under high serum condition; NS: not significant (genes with FDR > 0.05 when comparing heterospheroids with monospheroids either in high serum condition or in low serum condition); abs(log2FC): absolute log_2_ fold change.

**Supplemental Figure S5**

High serum DEGs compared with Bailey’s classification

High serum DEGs compared with Moffitt’s classification


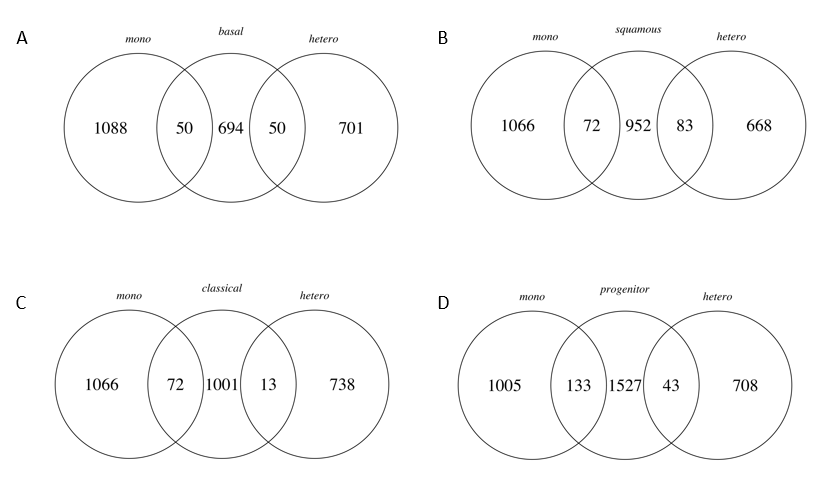


5.7%

11.7%

1.7%

6.3%

6.3%

11.1%

4.4%

6.7%

High serum DEGs compared with Bailey’s classification

Low serum DEGs compared with Bailey’s classification

Low serum DEGs compared with Moffitt’s classification

High serum DEGs compared with Moffitt’s classification


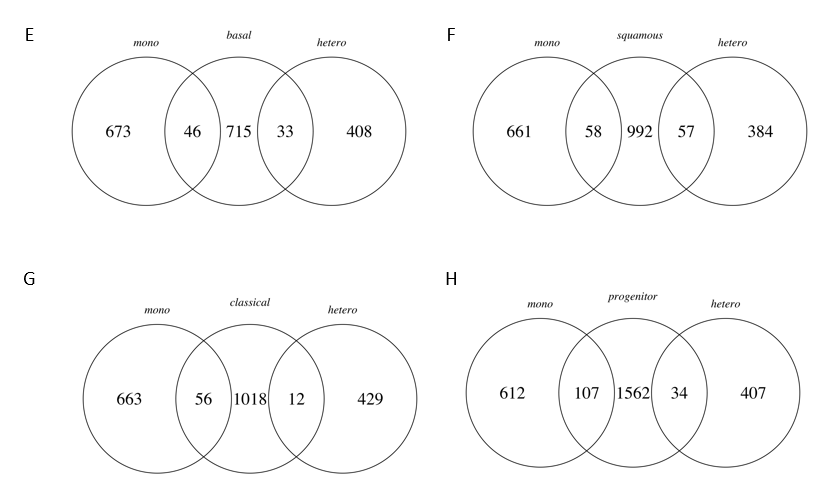


7.7%

14.9%

2.7%

7.8%

12.9%

8.1%

6.4%

7.5%

Low serum DEGs compared with Bailey’s classification

Low serum DEGs compared with Moffitt’s classification

**Supplemental Figure S5. Differentially expressed genes of Panc1 from spheroid model overlap with significantly changed genes from patients in the TCGA_PAAD cohort based on Moffitt’s classification and Bailey’s classification.**

High serum condition: A-D

(A) Venn diagram showing overlaps of differentially expressed genes (DEGs) with higher transcript levels of Panc1 grown in hetero- versus monospheroids (hereafter called “DEGs higher in heterospheroids”) and DEGs that had higher transcript levels for Panc1 grown in mono- versus heterospheroids (hereafter called “DEGs higher in monospheroids”) with DEGs that had higher transcript levels for the basal-like subtype TCGA patient group when compared to classical subtype (Overlap: 6.7% vs. 4.4%, *P* = 0.036) [1]. (B) Venn diagram showing overlaps of DEGs higher in heterospheroids and DEGs higher in monospheroids with DEGs had higher transcript levels for the squamous subtype TCGA patient group compared to the progenitor subtype TCGA patient group (Overlap: 11.1% vs. 6.3%, *P* = 3.07×10^-4^) [1]. (C) Venn diagram showing overlaps of DEGs higher in heterospheroids and DEGs higher in monospheroids with DEGs that had higher transcript levels for the classical subtype TCGA patient group compared to the basal-like subtype TCGA patient group (Overlap: 1.7% vs. 6.3%, *P* = 1.12×10^-6^) [1]. (D) Venn diagram showing overlaps among DEGs higher in heterospheroids and DEGs higher in monospheroids with DEGs that had higher transcript levels for the progenitor subtype TCGA patient group compared to the squamous subtype TCGA patient group (Overlap: 5.7% vs. 11.7%, *P* = 3.20×10^-5^) [1].

Low serum condition: E-H

(E) Venn diagram showing overlaps analogous to A (Overlap: 7.5% vs. 6.4%, *P* = 0.474). (F) Venn diagram showing overlaps analogous to B (Overlap: 12.9% vs. 8.1%, *P* = 8.42×10^-3^). (G) Venn diagram showing overlaps analogous to C (Overlap: 2.7% vs. 7.8%, *P* = 2.63×10^-4^). (H) Venn diagram showing overlaps analogous to D (Overlap: 7.7% vs. 14.9%, *P* = 2.82×10^-4^). *P* value was obtained through Fisher’s exact test.

**Supplemental Figure S6**

**
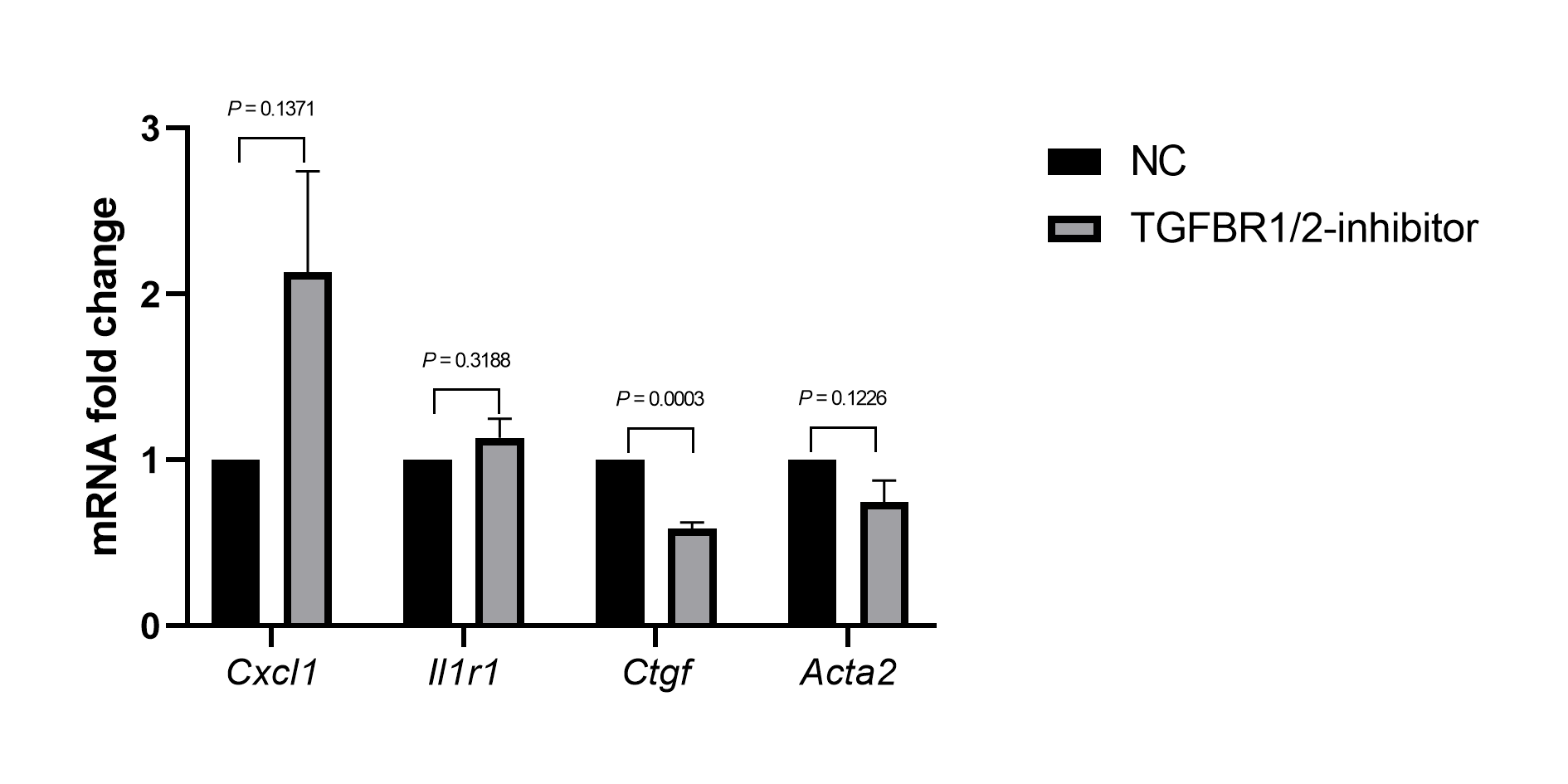
**

**Supplemental Figure S6. Expression of myCAF/iCAF marker gene changes following treatment with** **TGFBR1/2 kinase inhibitor.**

mRNA expression changes of myCAF marker genes *Ctgf* and *Acta2* and iCAF marker genes *Cxcl1* and *Il1r1* in mPSCs from heterospheroids treated with TGFBR1/2 kinase inhibitor under high serum condition. Bars show standard error of the mean (SEM).

**Supplemental Figure S7**

**
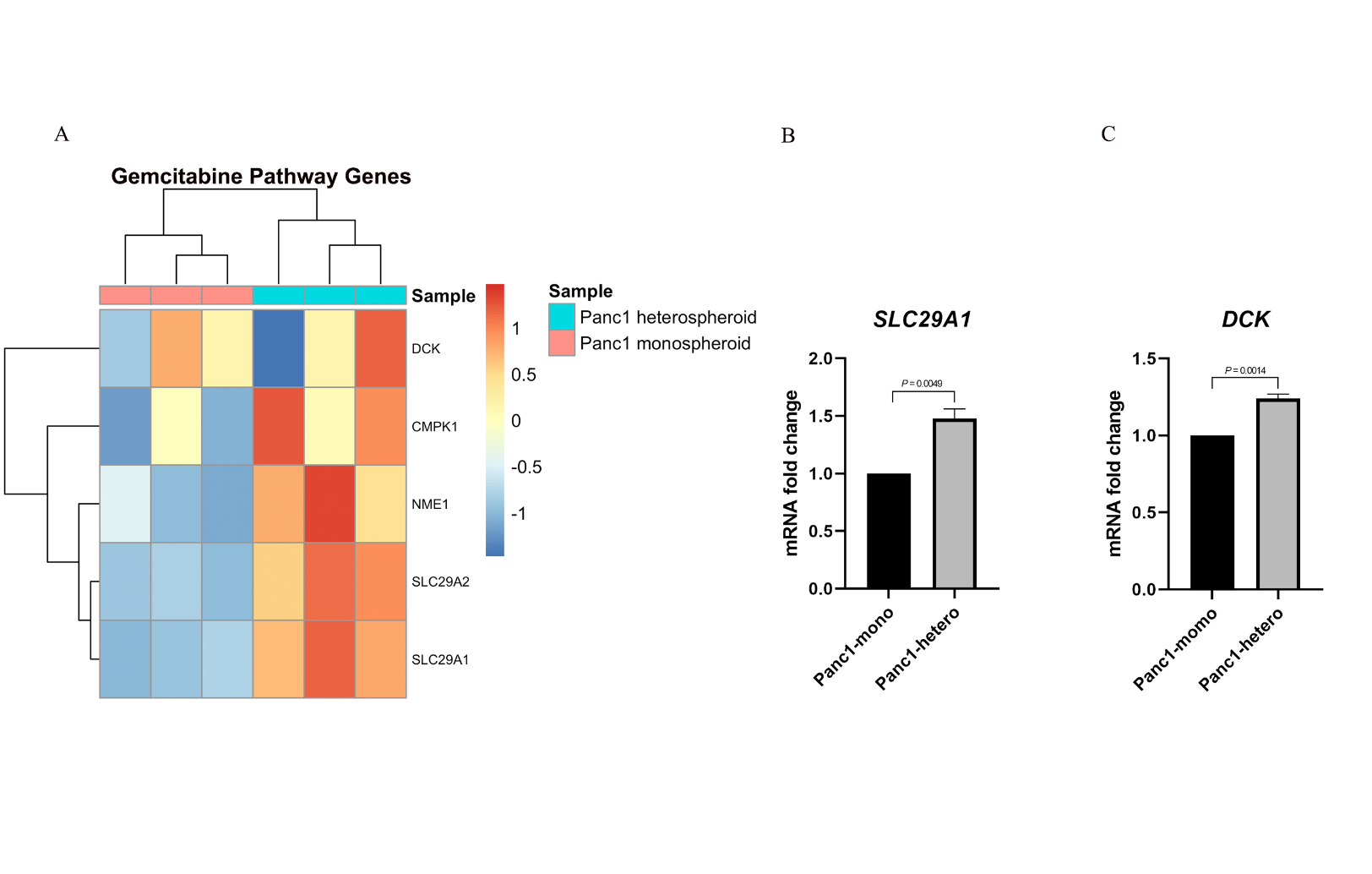
**

**Supplemental Figure S7. Expression of relevant genes for gemcitabine activity in Panc1 from monospheroids and heterospheroids under high serum condition.**

(A) Heatmap showing the relative transcript levels of gemcitabine transporter genes and metabolizing enzyme genes of Panc1 cells from monospheroids and heterospheroids. (B, C) mRNA expression of *SLC29A1* and *DCK* in Panc1 cells from monospheroids and heterospheroids analyzed by qRT-PCR. Bars show standard error of the mean (SEM).

Reference:

[1] (2017). Integrated Genomic Characterization of Pancreatic Ductal Adenocarcinoma *Cancer cell* **32**, 185-203.e113.

**Supplemental** **Table legends**

**Supplemental Table S1. List of species-specific primers used for qRT-PCR**

**Supplemental Table S2. Comparison of mRNA levels of Panc1 between heterospheroids and monospheroids under high serum condition.**

**Supplemental Table S3. Summary of gene set enrichment analysis for differentially regulated genes of Panc1 from heterospheroids compared to monospheroids under high serum condition.**

**Supplemental Table S4. Comparison of mRNA levels of mPSCs between heterospheroids and monospheroids under high serum condition.**

**Supplemental Table S5. Summary of gene set enrichment analysis for differentially regulated genes of mPSCs from heterospheroids compared to monospheroids under high serum condition.**

**Supplemental Table S6. Comparison of mRNA levels of Panc1 between heterospheroids and monospheroids under low serum condition.**

**Supplemental Table S7. Summary of gene set enrichment analysis for differentially regulated genes of Panc1 from heterospheroids compared to monospheroids under low serum condition.**

**Supplemental Table S8. Comparison of mRNA levels of mPSCs between heterospheroids and monospheroids under low serum condition.**

**Supplemental Table S9. Summary of gene set enrichment analysis for differentially regulated genes of mPSCs from heterospheroids compared to monospheroids under low serum condition.**
